# Lysis of *Escherichia coli* by colicin Ib contributes to bacterial cross-feeding by releasing active β-galactosidase

**DOI:** 10.1093/ismejo/wraf032

**Published:** 2025-02-19

**Authors:** Nicole A Lerminiaux, Jaycee M Kaufman, Laura J Schnell, Sean D Workman, Danae M Suchan, Carsten Kröger, Brian P Ingalls, Andrew D S Cameron

**Affiliations:** Institute for Microbial Systems and Society, Faculty of Science, University of Regina, Regina, Saskatchewan, S4S 0A2, Canada; Department of Biology, Faculty of Science, University of Regina, Regina, Saskatchewan, S4S 0A2, Canada; Department of Applied Mathematics, University of Waterloo, Kitchener-Waterloo, Ontario, N2L 3G1, Canada; Klick Applied Sciences, Klick Inc., Toronto; Institute for Microbial Systems and Society, Faculty of Science, University of Regina, Regina, Saskatchewan, S4S 0A2, Canada; Department of Biology, Faculty of Science, University of Regina, Regina, Saskatchewan, S4S 0A2, Canada; Institute for Microbial Systems and Society, Faculty of Science, University of Regina, Regina, Saskatchewan, S4S 0A2, Canada; Department of Biology, Faculty of Science, University of Regina, Regina, Saskatchewan, S4S 0A2, Canada; Institute for Microbial Systems and Society, Faculty of Science, University of Regina, Regina, Saskatchewan, S4S 0A2, Canada; Department of Biology, Faculty of Science, University of Regina, Regina, Saskatchewan, S4S 0A2, Canada; Department of Microbiology, School of Genetics and Microbiology, Moyne Institute of Preventive Medicine, Trinity College Dublin, Dublin D02A2H0, Ireland; Department of Applied Mathematics, University of Waterloo, Kitchener-Waterloo, Ontario, N2L 3G1, Canada; Institute for Microbial Systems and Society, Faculty of Science, University of Regina, Regina, Saskatchewan, S4S 0A2, Canada; Department of Biology, Faculty of Science, University of Regina, Regina, Saskatchewan, S4S 0A2, Canada

**Keywords:** colicin ib, cross-feeding, microbial community, β-galactosidase, bacteriocin

## Abstract

The diffusible toxin ColIb produced by *Salmonella enterica* serovar Typhimurium SL1344 is a potent inhibitor of *Escherichia coli* growth. To identify and parameterize metabolic cross-feeding in states of competition, we established defined communities in which *E. coli* was the only species able to access a sole carbon source, lactose. Although ColIb was predicted to undermine cross-feeding by killing the lactose-converting *E. coli*, *S. enterica* populations thrived in co-culture. We discovered that ColIb caused the release of active β-galactosidase from *E. coli* cells, which induced galactose uptake by *S. enterica*. Although iron limitation stimulates ColIb production and makes *E. coli* more sensitive to the toxin, ColIb killing in iron-limited conditions did not enhance iron acquisition or siderophore scavenging by *S. enterica*. Also unexpected was the rapid rate at which resistance to ColIb evolved in *E. coli* through spontaneous mutation of the ColIb receptor gene *cirA* or horizontal acquisition of the *S. enterica* colicin immunity gene *imm*. Mathematical modelling effectively predicted the growth kinetics of *E. coli* and *S. enterica* populations, revealing a tractable system in which ColIb can shrink a competitor population while simultaneously amplifying the metabolic contributions of the suppressed population.

## Introduction

Microbial community members are interconnected by the flow of metabolites as each cell modifies the environmental milieu by accessing specific nutrients, ejecting waste products, and releasing high-value compounds like enzymes and polymers. Released metabolites support a spectrum of symbiotic relationships, from cooperative to antagonistic [1]. A particular challenge for bacteria is that they often need to secrete diffusible digestive enzymes to degrade organic polymers into transportable subunits [2]. However, the lack of control over secreted enzymes and diffusible metabolites allows non-producers to exploit producers’ efforts [3]. This cost of cheaters can be offset by mutualist interactions in which reciprocal production of “public goods” reduce the costs of resource stealing [1]. Despite the benefit of mutualist interactions, mathematical modelling and several experimental systems suggest competition, not cooperation, fosters microbiome stability [4, 5].

Niche overlap creates competition for limiting resources in most ecosystems [6, 7]. In mammal-associated microbiomes the host can exert numerous controls over the microbial community composition, including modulating inter-bacterial competition [8]. For example, iron sequestration by a host inflammatory response can trigger deadly competition between *Escherichia coli* and *Salmonella enterica* by stimulating both bacteriocin toxin sensitivity in *E. coli* and production toxin colicin Ib (ColIb) and a cognate immunity protein (Imm) by *S. enterica* serovar Typhimurium SL1344 [9]. Iron depletion causes both *E. coli* and *S. enterica* to upregulate production of a colicin receptor and iron transporter, CirA, resulting in rapid killing of sensitive *E. coli* if colicin is present [9, 10].

Bacteriocins operate over restricted phylogenetic ranges because they obstruct essential cellular processes unless cells are equipped with cognate immunity proteins [11, 12]. Bacteriocin-sensitive populations are also those most likely to be in competition in a community [6, 7, 13], consistent with mathematical modelling that found a narrow spectrum of bacteriocin activity benefits producers by inhibiting the strongest ecological competitors [13]. Several observations suggest bacteriocin producers can additionally benefit by predation of bacteriocin-sensitive cells [14]. *Bacillus subtilis* was observed to release a killing factor that was proposed to liberate nutrients for sporulating cells [15], but the nutrients responsible for cross-feeding were not determined. Similarly, competition between lactic-acid bacteria was observed to cause the release of cell wall components and DNA from non-bacteriocin-producing populations [16], but cross-feeding on released nutrients was not directly tested.

**Figure 1:**
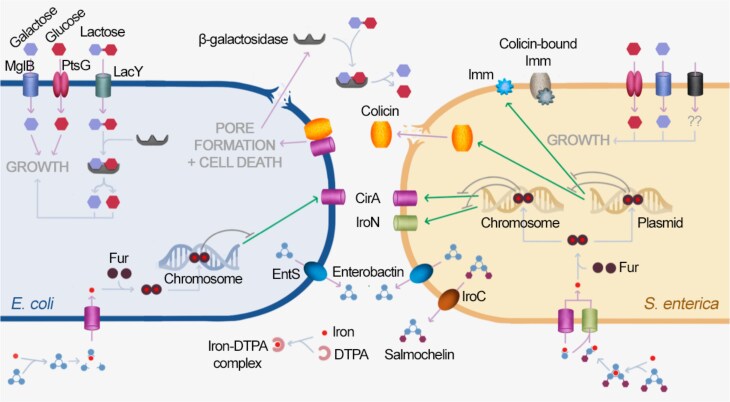
**Schematic diagram of colicin- and iron-mediated interactions between *E. coli* and *S. enterica* SL1344.** Intra- and extra-cellular metabolic pathways anticipated to contribute to species-species interactions in the synthetic two-species ecosystem are illustrated. When *E. coli* (left) and *S. enterica* (right) are co-cultured in an unstructured M9 + lactose environment, *E. coli* grows on lactose by importing lactose through the LacY transporter and β-galactosidase catalyzes the breakdown of lactose to glucose and galactose. *S. enterica* growth depends on metabolic byproducts from *E. coli*. The colicin ColIb produced by *S. Enterica* can bind to outer membrane iron transporter CirA. *S. enterica* CirA is protected from colicins by the plasmid-encoded immunity protein Imm. ColIb binding to *E. coli* CirA creates pores in the inner membrane, leading to loss of membrane potential and cell death. As demonstrated in the present study, β-galactosidase liberated by colicin attack can convert lactose to glucose and galactose in the culture supernatant. These sugar monomers serve as public goods that can be consumed by both *E. coli* and *S. enterica* through uptake via PtsG, MglB, and other transporters. ColIb and CirA levels are regulated according to iron availability (Fig. S1); when iron is scarce, the regulator fur is unable to bind to DNA, but when iron is abundant, fur represses both *cib* and *cirA* expression. *E. coli* and *S. enterica* produce siderophores to acquire iron, with both species secreting enterobactin via EntS and *S. enterica* additionally secreting salmochelin via IroC. Enterobactin acts as public goods because CirA can import enterobactin derivatives for both species, while salmochelin only serves *S. enterica*; *E. coli* is unable to import salmochelin because it does not encode the IroN importer. To simulate iron starvation, a chelator DTPA can be added to restrict iron availability for both species.

Comprehensive characterization of species-species interactions remains a significant challenge due to the complexity of ecological networks, particularly in species-rich microbial systems like those found in mammalian intestines. Synthetic communities provide an experimental alternative for parameterizing known and emergent properties of interactions. In a synthetic bacterial community where only *E. coli* can access a sole carbon and energy source (lactose), we found that ColIb caused the release of β-galactosidase from *E. coli*. The released enzyme was active in culture medium and allowed *S. enterica* to access galactose and glucose (Fig. 1). Mathematical modelling confirmed that the system is dominated by a small number of influential interactions.

## Materials and methods

### Bacterial strains and culture conditions

Strains and plasmids used and constructed in this study are listed in Supplementary Methods Table 1 and strain construction is described in supplementary text. *E. coli* W3110 contained plasmid pOT1 to enable gentamicin selection and is referred to as “*E. coli* WT” for simplicity in the text. A point mutation in *hisG* was selected in *S. enterica* SL1344 to restore ancestral histidine synthesis; this SL1344 derivative is referred to as “*S. enterica* SL1344 WT” for simplicity in the text. Culture media compositions are detailed in supplementary methods.

**Table 1:** Best fit parameter value estimates and uncertainty analysis (details in Methods).

**Parameter**	**Definition**	**Best-fit value**	**Units**	**Sensitivity**	**Identifiability score**	**95% confidence interval (%)**
${Y}_E$	Yield of *E. coli* growth	0.0393	[OD_600_/mM]	1670	1670	0.87
${Y}_S$	Yield of *S. enterica* growth on monosaccharide	2.52	[OD_600_/mM]	112	16.9	42
${k}_c$	Effectiveness of colicin (ColIb)	4.95	[OD_600_] ^-1^ hr^−1^	448	159	4.4
${k}_{LE}$	Rate of consumption of lactose by *E. coli*	4.73	[OD_600_]^−1^ hr^−1^	821	50.0	0.90
${k}_{BS}$	Rate of *S. enterica* growth on the byproduct (combined consumption rate and yield)	68.2	[OD_600_] ^-1^	660	336	0.90
${k}_{LG}$	Rate of conversion of lactose to monosaccharide	1.04	[OD_600_] ^-1^	58.0	25.4	46
${k}_{GS}$	Rate of consumption of monosaccharide by *S. enterica*	0.124	[OD_600_] ^-1^ hr^−1^	150	84.2	1.9
𝑝*_DTPA_*	[Fe] in the presence of 100 μM DTPA	0.425	[−]	120	118	1.25

### Polymicrobial culturing

All bacterial cultures described in this work were started fresh from a single colony streak isolated from −80°C freezer cultures to ensure clonal populations and to reduce the potential for contamination. Monocultures were grown in M9 + glucose (*E. coli*) or M9 + glycerol (*S. enterica*). In *E. coli* and *S. enterica* co-culture experiments, each strain was pre-grown overnight at 37°C with 200 rpm shaking in M9 + lactose (*E. coli*) or M9 + glucose (*S. enterica*) inoculated with a single colony. In the morning, cells were subcultured into fresh M9 medium with a starting OD_600_ = 0.075, followed by incubation at 37°C with 200 rpm shaking for 2–3 hours to reach OD_600_ = 0.1–0.2. Cultures were then passed through a 0.2 μm filter funnel (Thermo Fisher) with a vacuum to remove the supernatant and washed with pre-warmed carbon-free M9 medium. The filters were transferred to flasks containing 10 ml fresh pre-warmed M9 + lactose medium and were incubated at 37°C with 200 rpm shaking for 15 mins to dislodge cells from the filters. For co-culture experiments in flasks, cultures were combined to a starting OD_600_ = 0.05.

For plate reader experiments, cultures were combined to a starting OD_600_ = 0.0075 (about 1x10^6^ CFU/ml) unless stated otherwise, and this initial OD_600_ reading was determined with a Genesys 20 spectophotometer (Thermo Scientific, MA, USA). Optical density experiments were performed at 600 nm (OD_600_) in the SynergyHT Microplate Reader (BioTek, VT, USA). Reads were taken every 15 minutes over a minimum of 24 hours and cells were incubated at 37°C with constant shaking. 50 μl of light mineral oil was overlaid on 250 μl of culture in each well to prevent desiccation.

CFUs were determined by plating 10 μl drops of each step in a 10^−2^ to 10^−10^ 10-fold dilution series, with antibiotics in drop plates as appropriate.

### Co-culture RNA-seq

Cultures were pre-grown in M9 + glucose (*E. coli*) or M9 + glycerol (*S. enterica*), were vacuum filtered and washed to remove residual carbon sources, then were transferred into pre-warmed M9 + lactose at a starting density of 1x10^7^ CFU/ml. Monocultures and co-cultures were sampled immediately after filtering (0 h) and 6 hours later. Fixation, RNA extraction, rRNA depletion, cDNA synthesis and DNA sequencing were conducted as described in the supplementary methods. RNA-seq data was mapped to *Salmonella* and *E. coli* genomes using READemption (v1.0.5 [17]) with the “—crossalign_cleaning” option activated, which omits reads that map equally well to multiple locations in the reference sequences. All data reported in the manuscript are average transcripts per million (TPM) from two biological replicates. Differential gene expression comparisons and gene ontology (GO) analysis were conducted using the BioCyc Omics dashboard and transcriptomics functions with default settings [18, 19].

### Reverse transcriptase quantitative PCR

Quantitative PCR was conducted as in [20]. Details provided in supplementary methods.

### β-Galactosidase assay

Cultures were grown in M9 + lactose as described above and at 7 h, the supernatants were sampled for β-galactosidase using the Beta-Glo Assay System (Promega, WI, USA). Cultures were filter-sterilized through a 0.2 μm filter, and 100 μl of supernatant was added to 100 μl of reagent in a white-walled 96-well plate in triplicate. The plate was mixed and incubated in the dark for 30 mins at room temperature. Luminescence measurements were made with the SynergyHT Microplate Reader (BioTek, VT, USA), with an integration time of 0.5 s and gain set to 100. Background luminescence was subtracted from the readings and relative light units were normalized to the luminescence from the supernatant of *E. coli* monocultures.

### Preparation of cell-free spent media and colicin resistance assay

A colicin overlay on agar plates was used to calculate the percentage of *E. coli* that became resistant to colicin. To prepare cell-free medium containing colicins, stationary *S. enterica* SL1344 WT cultured in LB for 24 hours was centrifuged at 8000 × g for 10 minutes to pellet cells then the resulting supernatant was filter sterilized with a 0.2 μm syringe filter. For colicin+ LB agar plates, 200 μl of colicin-containing spent media was spread using glass beads and the plates were allowed to dry for 60 minutes before being used. Two additional cultures were started 6 hours and 24 hours after the first and processed in the same manner to have fresh spent media for each time point. For colicin-negative plates, 200 μl of plain LB was spread using glass beads and the plates were allowed to dry for 60 minutes before being used.

Monocultures and co-cultures began with inoculating 25 ml pre-warmed M9 + lactose in flasks with 0.0625 OD units of *E. coli* WT or 0.0625 OD units of each of *S. enterica* SL1344 WT and *E. coli* WT, respectively. Immediately after inoculation, after 6 hours of growth, and after 24 hours of growth, samples were removed, diluted in M9 + lactose, and 50 μl was spread plated on LB agar +/− colicins supplemented with 20 μg/ml gentamicin in triplicate. CFU/ml for each timepoint was calculated from manual colony counts and dilution factors.

### Whole genome sequencing and library preparation

DNA sequencing was conducted as in [21]; details provided in supplemental methods.

### Spot assays of colicin activity

Overnight *E. coli* WT cultures were diluted to McFarland turbidity standard = 2.0 (OD_600_ = 0.242) and 300 μl of the cell culture was spread on LB agar containing gentamicin. 10 μl of filter-sterilized spent medium from stationary *S. enterica* SL1344 WT in LB were dropped on the inoculated plates in triplicate and plates were incubated at 37°C for 24 h.

### β-Galactosidase cross-feeding assay

*E. coli* and *S. enterica* strains were grown to early stationary phase in shaking flasks (250 RPM) in LB (half sodium; 5.0 g/L NaCl), 100 μl of 10 mM DTPA, and 100 μl of 0.8 M IPTG. Stationary *E. coli* and either *S. enterica* 1344 WT or *S. enterica* SL1344 cib were added in a 1:1 ratio to a final volume of 25 ml then co-cultured for 4 hours with shaking at 37°C. No fresh LB was added, but 50 μl of 10 mM DTPA and 50 μl of 0.8 M IPTG was added. Co-cultures were filter sterilized using 0.2 μm filter cups (Thermo Scientific) into sterilized vacuum flasks. Filtered media was plated on LB agar plates to ensure sterility. Five ml of spent media were inoculated in tubes with stationary *S. enterica* SL1344 WT that was washed by centrifugation and resuspension in spent media. Where specified in the text, lactose (0.275 mM) or glucose (0.55 mM) were added to spent media immediately before addition of cells. Tubes were angled at 45 degree and shaken at 250 RPM at 37°C.

### Statistical analyses

All data was log-transformed prior to analysis to approximate normal (Gaussian) distribution. Statistical analyses were performed with GraphPad Prism version 9.3.1. Depending on the analysis, the one-way analysis of variance (ANOVA) test was used, or two-way ANOVA test followed by either Tukey’s test for multiple comparisons (comparing all pairwise interactions) or Šídák’s test for multiple comparisons (comparing independent groups). *P* values less than 0.05 were considered significant.

### Model Calibration and uncertainty analysis

Development of the mathematical model is detailed in supplementary materials and methods. Model calibration, uncertainty analysis, along with calculation of sensitivity coefficients, identifiability scores and confidence intervals are described in Supplementary Methods.

## Results

### Cross-feeding and antagonism between bacterial species in a lactose-rich environment

**Figure 2:**
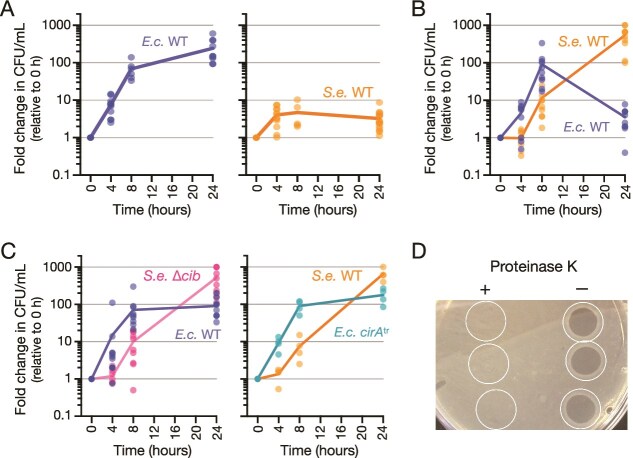
**Cross-feeding and competition between bacterial species in a lactose-rich environment.** (**A**) Growth curves plotting fold-change in cell numbers over 24 hours for monocultures of *E. coli, E.c.*, and *S. enterica*, *S.e.*, in M9 + lactose in shaking flasks. Cultures were started at 1x10^6^ CFU/ml in M9 + lactose. (**B & C**) Co-cultures conducted in 250 μl in a shaking plate reader in M9 + lactose. Data plotted as fold-change in *E.c.* and *S.e.* cell numbers over 24 hours in co-culture. *E.c. cirA*^tr^ is insensitive to colicin, and *S.e.* Δ*cib* does not produce colicin. (**D**) Spot assay of *S.e.* SL1344 cell-free spent media confirmed that the molecule inhibiting *E.c.* growth is a protein. Spent medium was treated with (left) or without (right) proteinase K before spotting onto a lawn of *E.c.* WT cells. Plates were incubated for 24 hours at 37°C.

Synthetic bacterial communities composed of select species in defined media enable quantitative analyses of ecological relationships [22]. To discover and characterize interactions between model organisms, we constructed a metabolic dependency in a defined community of *Escherichia coli* MG1655 and *Salmonella enterica* serovar Typhimurium SL1344 (*S. enterica* SL1344). Tracking population sizes in co-cultures by counting colony forming units (CFU) confirmed that *E. coli* population growth was robust in a lactose-containing minimal medium (M9 + lactose), but monoculture of *S. enterica* SL1344 inoculated with exponentially growing cells stopped increasing in abundance soon after transfer to M9 + lactose (Fig. 2A, S2A). In co-culture, *S. enterica* SL1344 could feed on *E. coli* metabolites to increase in population size over 600-fold (Fig. 2B). However, in co-culture *E. coli* exhibited a statistically significant 100-fold lower population size after 24 hours compared to growth in monoculture (Fig. 2B, S2A). *S. enterica* population growth in co-culture was equally robust in shaking flasks and in small volumes in a shaking plate reader (Fig. 2B, S2), though CFU counts showed that *S. enterica* populations experienced a small lag phase in plate reader assays (Fig. 2B).

### ColIb inhibits *E. coli* growth in co-culture

*S. enterica* SL1344 naturally contains a plasmid (pCol1B9) that encodes ColIb gene (*cib*) and cognate immunity gene (*imm*) [23]. The pore-forming colicin ColIb kills *E. coli* in rich medium (LB broth) and inhibits *E. coli* population growth in mouse intestines [10]. *E. coli* demonstrated uninhibited population growth and persistence when co-cultured with the non-colicin-producing *S. enterica* SL1344 Δ*cib/imm* mutant (Fig. 2C). We conducted a reciprocal test in *E. coli* carrying a non-functional truncated version of *cirA* (noted *cirA*^tr^), which alleviates sensitivity to ColIb. *E. coli cirA*^tr^ grew normally in the absence or presence of ColIb (Fig. 2C, S2A), confirming that ColIb activity explains the inhibition of *E. coli* growth in lactose minimal medium (Fig. S3). In M9 + lactose culture conditions, *E. coli* supported *S. enterica* population growth regardless of the presence or absence of colicin killing (Fig. 2B, C).

In M9 + glucose, *S. enterica* could grow without cross-feeding, yet *S. enterica* SL1344 continued to repress *E. coli* population growth (Fig. S2B). To test whether competition manifested through additional, non-colicin-mediated interactions, *E. coli* was co-cultured with *S. enterica* serovars lacking colicin genes: Typhimurium 14 028 s or Enteritidis EN1660 [24]. *E. coli* populations were stable in co-culture with alternate *S. enterica* strains (Fig. S2C), consistent with antagonism arising exclusively from colicin production by *S. enterica* SL1344. *S. enterica* spent medium (cell-free supernatant) contained ColIb that retains growth inhibitory properties when spotted on a lawn of *E. coli* (Fig. 2D). Treating the colicin-containing spent medium with proteinase K before spotting completely abolished growth inhibition, further confirming that the colicin is sufficient to explain inhibition of *E. coli* growth (Fig. 2D).

### Transcriptional responses to co-culture implicate hexose consumption by *S. enterica*

**Figure 3:**
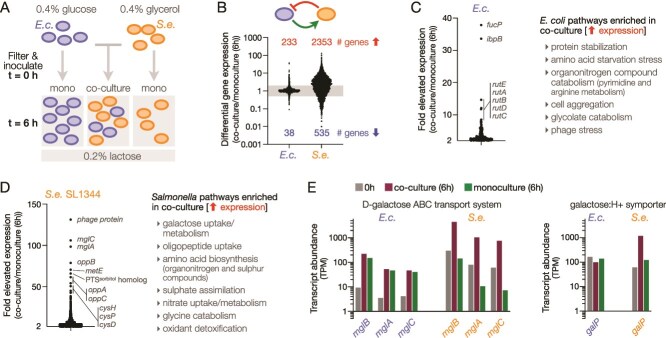
**Transcriptional responses to cross-feeding in co-culture**. (**A**) RNA-seq experimental design. Monocultures of *E. coli* (*E.c.*) and *S. enterica* (*S.e.*) were pre-grown in glucose or glycerol then were filtered and inoculated into fresh M9 + lactose at 1 × 10^7^ CFU/ml, and sampled for RNA-seq at 0 hour. Monocultures and co-cultures were sampled and fixed for RNA-seq at 6 h. two biological replicates were conducted for each condition and each time point. (**B**) Schematic representing the interactions involved in the two-species community. *E.c.* releases nutrients into the culture supernatant that *S.e.* can consume, while *S.e.* produces toxic colicins that inhibit growth of *E.c*. Differential gene expression was calculated by dividing TPM at 6 hours in co-culture by 6 hours in monoculture. Data points for all genes are plotted for each species. The shaded horizontal band indicates the range between 2-fold increased and 2-fold decreased expression in co-culture versus monoculture. The number of genes elevated or decreased greater than 2-fold in co-culture are indicated above and below the data points, respectively. (**C & D**) plots showing the most differentially expressed *E.c.* (C) and *S.e.* SL1344 (D) genes that were elevated in co-culture, as in (B) but here plotted on a linear y-axis. Gene ontology (GO) analysis identified biological processes elevated in co-culture. (**E**) Transcript abundances of galactose uptake genes in co-cultures and monocultures. Transcript abundances are reported in transcripts per million (TPM).

We applied RNA-seq for a genome-wide view of each species’ response to the co-culture conditions. Exponentially growing cells were inoculated into a mixed co-culture at a 1x10^7^ CFU/ml density (Fig. 3A). The starting population density was deliberately higher than conditions in Fig. 2 to intensify interspecies interactions early in culture growth while also providing capacity for multiple generations of growth in co-culture. To discern each species’ response to co-culture, each gene’s transcriptional output at 6 hours of co-culture was divided by its output at 6 hours in monoculture. *E. coli*’s transcriptome of 4382 genes was largely unaffected by co-culture (Fig. 3B), with only 233 genes increased greater than 2-fold and only 38 genes decreased by over 2-fold. In contrast, in *S. enterica* in co-culture, 2353 genes were more highly expressed than in monoculture at 6 h. Induction of over half of its complement of 4751 genes is consistent with its ability to grow and multiply by consuming *E. coli*-generated metabolites. Examining the most highly elevated genes in co-culture compared to monoculture further highlighted differences between each species’ cellular response. For example, in *E. coli*, protein stabilization (*ibpAB*), nitrogen scavenging from nucleotides (*rutABCDEFG*), and fucose uptake (*fucP*) were much more highly expressed in co-culture (Fig. 3C). *S. enterica* demonstrated a large number of differentially expressed genes in co-culture. Galactose uptake (*mglBAC*, *galS*), monosaccharide uptake (*ompC*, *ompF*, PTS sorbitol homologs), oligopeptide uptake (*oppABCD*), and sulfate transport and assimilation (*cysPUWAM*, *cysDNC*, *cysJIH*, *cysK,* and *cysZ*) were the most elevated (Fig. 3C). The upregulation of amino acid biosynthesis, peptide uptake, and nitrogen and sulfur scavenging reflect the favourable growth conditions for *S. enterica* in co-culture (Fig. 3C).

Galactose uptake by the ABC transporter MglBAC was the most differentially expressed process in *S. enterica* in co-culture (Fig. 3D, E). In contrast, *E. coli mglBAC* expression was equivalent between co-culture and monoculture at 6 hours (Fig. 3E). There was a striking similarity in transcriptional output of *S. enterica mglBAC* in glycerol (t = 0 h) and *E. coli mglBAC* at 6 hours (Fig. 3E), conditions when CRP is active, which may represent the baseline CRP-activation of the *mglBAC* operon in both species. Thus, increased *E. coli mglBAC* transcription at 6 hours relative to pre-culture in glucose (t = 0 h) likely reflects activation of the CRP regulon by the loss of glucose. *E. coli*’s strongest transcriptional response in both monoculture and co-culture was induction of the *lacZYA* operon for lactose uptake and metabolism (Fig. S4A). The similarly high transcriptional output of *S. enterica mglBAC* in co-culture could also reflect full activation in response to extracellular galactose. Transcription of the glucose and galactose:H+ symporter encoded by *galP* differed between the two species in co-culture (Fig. 3E). *galP* expression was unchanged in *E. coli* across all conditions, whereas the gene was strongly induced in *S. enterica* in co-culture. Additional monosaccharide uptake systems that were differentially expressed between *E. coli* and *S. enterica*, *fucP* and PTS^sorbitol^ (Fig. 3C, D), are plotted in Fig. S4.

Galactose metabolism was expected in *E. coli* populations in monocultures and co-cultures because the populations were actively metabolizing lactose as a sole carbon and energy source. However, it was surprising to observe galactose metabolism in *S. enterica* because acetate and glycerol have been experimentally established to cross-feed subpopulations of *E. coli* in co-culture [7, 25–28]. It is important to note that despite the strong upregulation of galactose uptake by *S. enterica* in co-culture with colicin, Fig. 2C shows that *S. enterica* received sufficient nutrients for growth in the absence of colicin.

### ColIb liberates active β-galactosidase from *E. coli*

**Figure 4:**
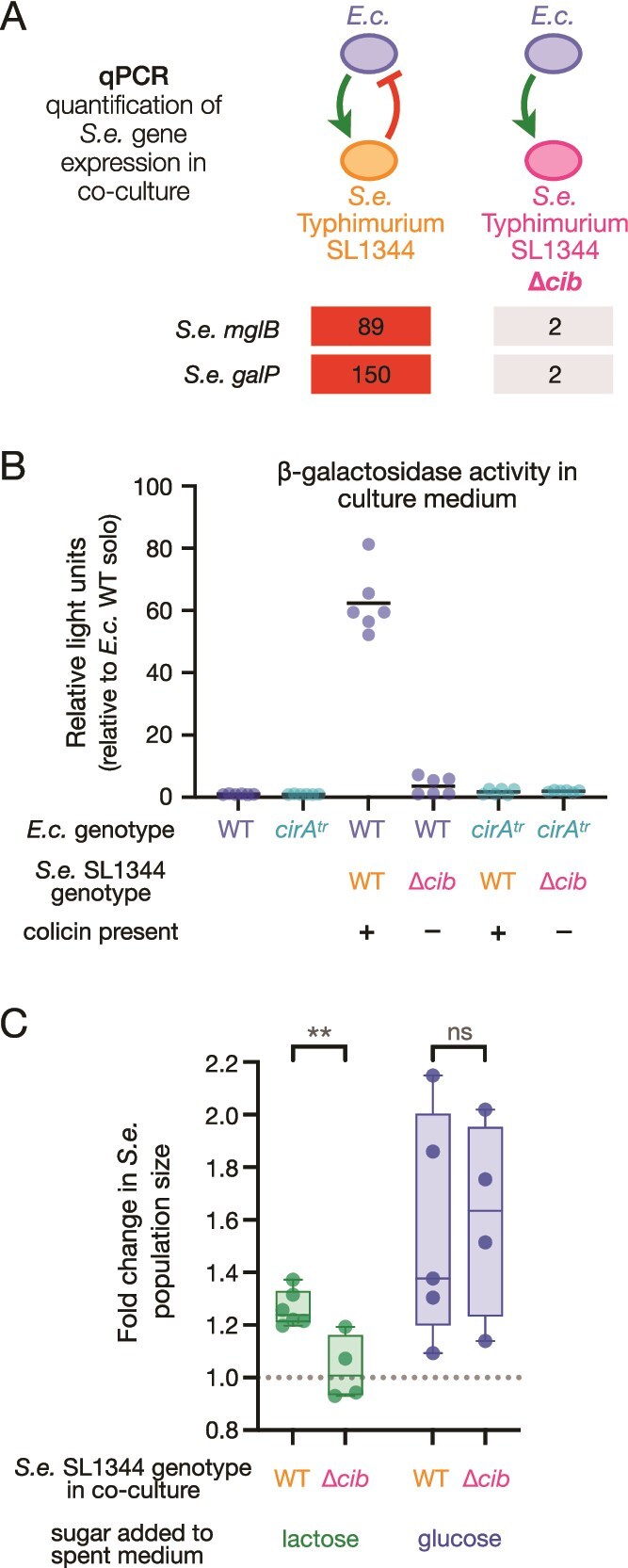
**Colicin attack improves sugar availability for *Salmonella*.**(**A**) Fold-change in relative transcript abundance of galactose uptake genes *mglB* and *galP* in *S.e.* WT and *S.e.* Δ*cib* when co-cultured with *E.c.* WT. Each strain was started at 1x10^7^ CFU/ml in M9 + lactose and RNA samples were taken at 0 and 5 hours. qPCR quantification is the result of averaging two technical replicates. Transcript values were quantified from a relative standard curve and normalized to transcripts at 0 hour. The line represents the mean of two biological replicates. (**B**) β-galactosidase activity measured in cell-free culture supernatant.Relative light units (RLU) were measured using a plate reader and normalized by the amount of viable *E.c.* CFUs determined by plate counts. All cultures were grown for 7 hours in M9 + lactose before being filtered. The cell-free supernatant was added to an equal volume of luminescent reagent. The mean of six trials is plotted for each condition, with three biological replicates conducted on two separate days. A two-way ANOVA was performed to analyze the effect of strain and culture condition (2-species community and monoculture) on enzymatic activity. There was a statistically significant difference in enzymatic activity by strain (F (1, 30) = 78.03, *p* < 0.0001), by culture condition (F (2, 30) = 78.47, *p* < 0.0001), and by interaction between strain and culture condition (F (2, 30) = 58.41, *p* < 0.0001). Tukey’s test for multiple comparisons revealed significant pairwise differences in enzymatic activity when *E.c.* WT was co-cultured with *S.e.* SL1344 WT and all other conditions (p_adj_ = <0.001). Significant pairwise differences were also detected between monoculture *E.c.* WT and co-culture of *E.C.* WT and *S.e.* Δ*cib* (*p* = 0.0096), and monoculture *E.c. cirA*^tr^ and co-culture of *E.C.* WT and *S.e.* Δ*cib* (*p* = 0.0051). No other comparisons were statistically significant. (**C**) Wildtype *S.e.* was inoculated into spent (filtered) LB in which stationary phase *E.c.* and *S.e.* were co-cultured in the presence of EDTA to enhance colicin production and sensitivity. *S.e.* Did not benefit from added lactose if the co-culture condition contained the colicin deficient mutant *S.e.* Δ*cib*. *S.e.* consistently benefited from added glucose regardless of the colicin status of the co-culture. Values are calculated relative to *S.e.* growth in the absence of added sugar. Unpaired t-test was used to test for statistical differences (^**^ = *P* value 0.005).

We hypothesized that colicin attack causes *E. coli* cells to release β-galactosidase, in turn making more nutrients accessible for *S. enterica* SL1344. We used quantitative PCR (qPCR) to further interrogate the transcriptional response of *S. enterica*’s high-affinity galactose transporter *mglB* and low-affinity galactose transporter *galP* in co-cultures with and without colicin. Transcription of *mglB* and *galP* was elevated in co-cultures with *S. enterica* SL1344 WT compared to *S. enterica* SL1344 Δ*cib* (Fig. 4A), confirming that the presence of ColIb increased the availability of galactose in the culture medium.

We next directly quantified β-galactosidase enzymatic activity in culture supernatants [29, 30]. Relative light units were normalized to the number of viable *E. coli* CFU/ml. Low levels of luminescence were observed in all conditions where ColIb is either not present or rendered ineffective: *E. coli* monoculture, co-culture of *E. coli* and *S. enterica* SL1344 Δ*cib*, and co-culture of ColIb-insensitive *E. coli cirA*^tr^ and *S. enterica* SL1344 WT (Fig. 4B). Co-culture of *S. enterica* SL1344 WT and *E. coli* WT produced statistically significant higher relative light units (Fig. 4B), directly implicating the release of β-galactosidase by ColIb attack on *E. coli*.

As noted above, *S. enterica* SL1344 achieved the same cell numbers after 24 hours regardless of the presence or absence of colicin (Fig. 2B), indicating that cross-feeding relies on *E. coli* overflow metabolism. To test whether lactose catabolism by extracellular β-galactosidase contributes to growth, we tested growth potential in spent LB medium, which lacks fermentable sugars, acetate, pyruvate, and glycerol [31]. Parallel experiments were not conducted in spent M9 minimal medium because culture ODs declined upon inoculation of spent minimal media, which complicated data interpretation (not shown). To test whether released β-galactosidase enhances growth on lactose, stationary phase monocultures were mixed for 2 hours to enable lysis, then were filter sterilized so that spent media contained released β-galactosidase. Spent media were then inoculated with *S. enterica* SL1344 WT and growth was measured in the presence or absence of a hexose sugar. When lactose was added to spent media, *S. enterica* demonstrated consistently greater growth in spent media that contained colicin (*E. coli* WT and *S. enterica* SL1344 WT) compared to spent media lacking colicin (*E. coli* WT and *S. enterica* SL1344 Δ*cib*) (Fig. 4C). The addition of glucose generated variable growth in spent media, yet there was no difference in average growth between spent media from co-culture with colicin compared to spent media from co-culture lacking colicin. This data connects the observed β-galactosidase activity (Fig. 4B) to an enhanced growth potential for *S. enterica* if spent media contains β-galactosidase liberated by colicin (Fig. 4C).

### ColIb does not improve iron availability for *S. enterica*

*E. coli* and *S. enterica* both produce siderophores that can be poached by neighbouring cells (Fig. 1) [32, 33], resulting in significant niche overlap and resource stealing in competition for iron. *S. enterica* produces two siderophores, enterobactin, and salmochelin, whereas *E. coli* produces enterobactin but cannot access iron bound to salmochelin [34]. The increased production of colicins during iron starvation has been hypothesized to liberate iron for a colicin-producing population [35, 36]; thus, we predicted that *S. enterica* would have a competitive advantage in our synthetic ecosystem through lytic release of *E. coli*’s cellular iron plus exclusive access to salmochelin (Fig. 1). *cib* expression was elevated in the *S. enterica* enterobactin (*ΔentA*) mutant compared to wildtype, consistent with the enterobactin mutant responding to more severe iron limitation due to reduced scavenging (Fig. S5A). However, the expression of siderophore synthesis genes (*entA* for enterobactin, *iroB* for salmochelin) changed very little in the presence or absence of ColIb (Fig. S5B).

**Figure 5:**
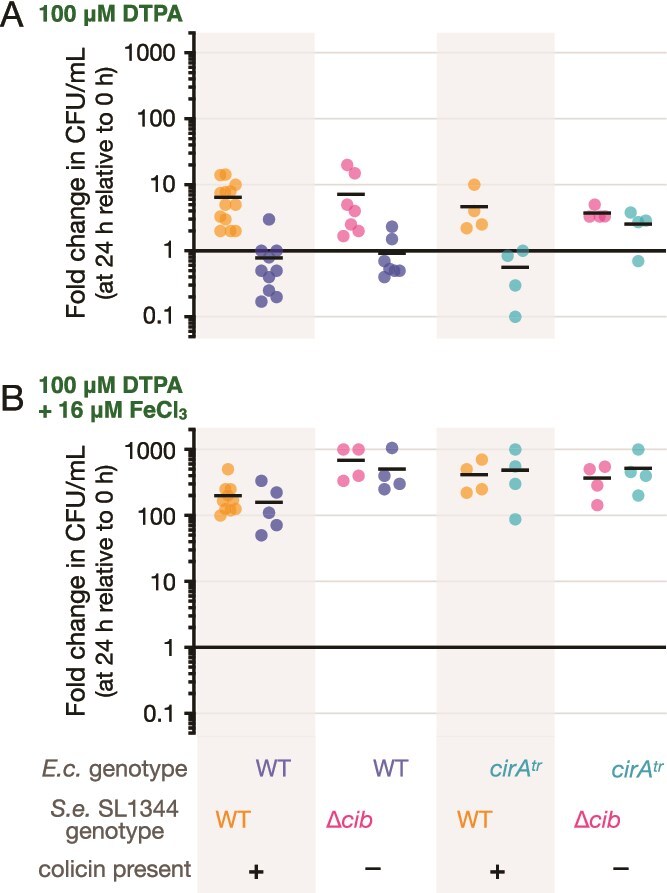
**DTPA limits growth of co-cultures, which can be rescued by iron supplementation.** (**A**) Severely iron limited conditions were created by addition of 100 μM DTPA to M9 + lactose minimal medium, into which *E.c.* and *S.e.* were inoculated at equal densities of 10^6^ CFU/ml. Fold-change in abundance at 24 hours is expressed relative to 0 hour. Each point is a biological replicate (n = 4–10) and horizontal bars represent means. A two-way ANOVA was performed to analyze the effect of strain and culture condition (2-species community) on *E.c.* abundance. There was a statistically significant difference in abundance by culture condition (F (1, 21) = 7.793, p = 0.0109), though strain effect and interaction terms were not significant. Tukey’s test for multiple comparisons revealed significant pairwise differences in *E.c.* abundance between *E.c.* WT co-cultured with *S.e.* SL1344 WT and *E.c. cirA^tr^* co-cultured with *S.e.* SL1344 Δ*cib* (p_adj_ = 0.0492), and between *E.c. cirA^tr^* co-cultured with *S.e.* SL1344 WT and *E.c. cirA^tr^* co-cultured with *S.e.* SL1344 Δ*cib* (p_adj_ = 0.0440). No other comparisons were statistically significant. A second two-way ANOVA was performed to analyze the effect of strain and culture condition (2-species community) on *S.e.* abundance, but no significant differences were detected. (**B**) Adding iron to DTPA-treated co-cultures restores *E.c.* growth in lactose minimal medium. M9 + lactose culture medium was prepared with 100 μM DTPA and 16 μM FeCl_3_ followed by inoculation of *E.c.* and *S.e.* at equal densities of 10^6^ CFU/ml. Each point is a biological replicate (n = 4–10) and lines represent the mean. A two-way ANOVA was performed to analyze the effect of strain and culture condition on *E.c.* abundance, but no significant differences were detected. A second two-way ANOVA was performed to analyze the effect of strain and culture condition (2-species community) on *S.e.* abundance. There was a statistically significant difference in abundance by strain (F (1, 18) = 4.809, p = 0.0417), and interaction between strain and culture condition (F (1, 18) = 7.503, p = 0.0135), though culture condition alone was not significant. Tukey’s test for multiple comparisons revealed significant pairwise differences in *S.e.* abundance between *S.e.* SL1344 WT co-cultured with *E.c.* WT and *S.e.* SL1344 Δ*cib* co-cultured with *E.c.* WT (p_adj_ = 0.0060). No other comparisons were statistically significant.

In the defined medium M9, iron is present as a contaminant from chemical ingredients and glassware; this contamination is estimated to provide iron concentrations of 0.15–1.8 *μ*M [37–40]. To examine how ColIb production responded to iron manipulation, *cib* transcript abundance in *S. enterica* was measured over time in the presence of the iron chelator DTPA and iron supplementation. In rich medium, addition of excess DTPA (100 μM) resulted in elevated ColIb production by *S. enterica* SL1344 and a mass killing of *E. coli* (Fig. S6), as previously reported [9]. Addition of 100 μM DTPA to M9 minimal medium strongly inhibited both *E. coli* and *S. enterica* SL1344 population growth in co-culture in M9 + lactose (Fig. 5A). Iron-starved cells remained viable, as determined by plate counts of colony forming units, but removing colicin production from the system had no statistically significant effect on growth of *E. coli* WT (Fig. 5A), indicating that iron starvation, not ColIb levels, explained the arrest in community growth. Adding iron to the DTPA-treated cultures at a concentration of 16 μM restored population growth for both species and removed ColIb-mediated suppression of *E. coli* (Fig. 5B). In all co-culture combinations with iron supplementation, both *E. coli* and *S. enterica* benefited when antagonism was removed by deleting *cib* or *cirA* (Fig. 5B).

### Development of a mathematical model of population dynamics

**Figure 6:**
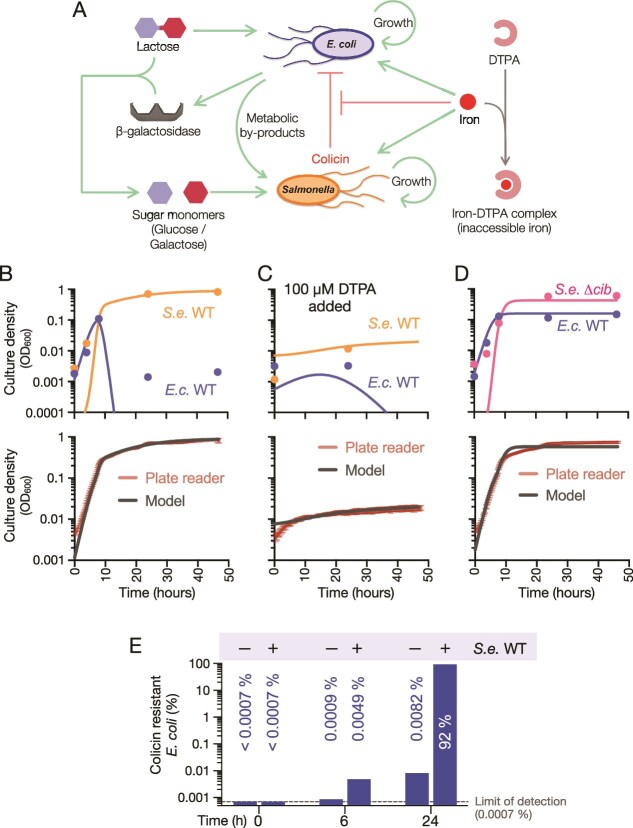
**The mathematical description of the co-culture system.** (**A**) Interaction diagram of the two-species community captured by the mathematical model. *E. coli* (*E.c.*) growth is dependent on lactose availability. *S. enterica* (*S.e.*) consumes unidentified metabolites produced by *E.c.* and sugar monomers produced by β-galactosidase. β-galactosidase is released from *E.c.* cells lysed by colicin activity. Iron contributes to the growth of both species; iron represses colicin release and sensitivity. The chelator DTPA can sequester iron when present in co-culture, rendering iron unavailable for both species. In the model formulation, levels of iron and DTPA are presumed fixed, whereas colicin and β-galactosidase levels are tied directly to the producing species concentrations. (**B-D**) Experimentally observed single population and whole community abundances compared to model predictions in three scenarios. These are randomly chosen representatives from each scenario; the remaining replicates from each scenario are found in Fig. S7. (**B**) *S.e.* SL1344 WT and *E.c.* WT co-culture, (**C**) *S.e.* SL1344 WT and *E.c.* WT co-culture with 100 *μ*M DTPA and (**D**) *S.e.* SL1344 *Δcib* and *E.c.* WT co-culture. Each point in B, C, D is a biological replicate (n = 4–6) and error bars represent standard deviation. Total population counts were determined as OD_600_ measurements. For mixed cultures, strain-selected CFU counts were then used to determine the strain population ratios. Error bars on the plate reader lines represent standard deviation. Model parameters are described in Table 1. (**E**) Percentage of colicin-resistance *E.c.* at 0, 6 , and 24 hours when growing monoculture or with *S.e.* SL1344 WT. The assay limit of detection was less than 0.0007%.

Synthetic communities facilitate quantitative assessment of community composition and metabolic inputs, allowing for mathematical modelling to interrogate the relative contributions of observed and hidden interactions between species; e.g. see references [41–43]. We developed an ordinary differential equation model that describes the dynamics of the two interacting bacterial species, with species abundance (in units of OD_600_) represented by *E*(*t*) (*E. coli*) and *S*(*t*) (*S. enterica*), and concentrations of lactose (*L*(*t*)) and monosaccharides (*G*(*t*), a combined pool of galactose and glucose) in units of mM. We began our model development with a description that captured all interactions between wildtype *E. coli* and wildtype *S. enterica* in our co-culture ecosystem (Fig. 1). A model reduction process based on fits against preliminary datasets led us to a final model formulation consisting of four equations involving seven kinetic parameters that capture the simplified system presented in Fig. 6A. The equations are as follows:


(1) \begin{equation*}\kern-3.6pc \frac{d}{dt}E(t)={Y}_E{k}_{LE} FeL(t)E(t)-\frac{k_cE(t)S(t)}{Fe^2} \end{equation*}

(2) \begin{equation*} \frac{d}{dt}S(t)={Y}_S{k}_{GS} FeG(t)S(t)+{k}_{BS}{Y}_E{k}_{LE} FeL(t)E(t)S(t) \end{equation*}

(3) \begin{equation*}\kern-2.5pc \frac{d}{dt}L(t)=-{k}_{LE}L(t)E(t)-{k}_{LG}L(t)\frac{k_cE(t)S(t)}{Fe^2} \end{equation*}

(4) \begin{equation*}\kern-2.5pc \frac{d}{dt}G(t)=2{k}_{LG}L(t)\frac{k_cE(t)S(t)}{Fe^2}-{k}_{GS}S(t)G(t) \end{equation*}

The first term in Equation 1 captures *E. coli* dynamics. The *E. coli* growth rate is jointly proportional to the relative iron concentration $Fe$ (unitless) and the abundance of lactose. Parameter ${k}_{LE}$ characterizes growth rate; ${Y}_E$ is a growth yield coefficient. The influence of monosaccharide on the rate of *E. coli* growth was found to be negligible in preliminary model development. The second term represents colicin-induced cell lysis. This rate is inversely proportional to (i) expression of the colicin receptor *cirA* by *E. coli* and (ii) expression of colicin by *S. enterica*. Both these processes are induced by iron, leading to the square term in the denominator. The abundance of colicin is assumed proportional to the *S. enterica* population size. Parameter ${k}_c$ captures the combination of this proportionality and the colicin-induced death rate.

Equation 2 describes the *S. enterica* population dynamics. The first term represents growth on monosaccharide (again, jointly proportional to iron level). Here parameter ${k}_{GS}$ captures the rate of growth of *S. enterica* on monosaccharide and ${Y}_S$ is a growth yield coefficient. The second term represents growth on an unspecified by-product of *E. coli* metabolism (with abundance assumed proportional to the *E. coli* growth rate). Parameter ${k}_{BS}$ captures the rate of growth of *S. enterica* on this metabolic byproduct. No appreciable loss of *S. enterica* was observed in the experiments, and so no elimination term was included in the model.

Equation 3 captures lactose dynamics. The first depletion term describes the rate of uptake by *E. coli*. The second term reflects breakdown of lactose into monosaccharide by β-galactosidase, which is presumed proportional to the rate of *E. coli* lysis. Parameter ${k}_{LG}$ captures the rate of conversion of lactose to monosaccharide.

Finally, Equation 4 represents the concentration of monosaccharide production from lactose via β-galactosidase (with a two-to-one stoichiometry) and consumption by the *S. enterica* population.

We calibrated the model against optical density data from plate reader co-cultures and CFU count data of individual species from three experimental scenarios (representative data in Fig. 6, remaining datasets in Fig. S7): (i) co-culture of wildtype *E. coli* and *S. enterica* in M9 + lactose (Fig. 6B, S7A); (ii) co-culture of wildtype *E. coli* and *S. enterica* in M9 + lactose, with 100 μM DTPA (Fig. 6C, S7B); and (iii) co-culture of colicin-mutant *S. enterica* SL1344 Δ*cib* and wildtype *E. coli* in M9 + lactose (Fig. 6D, S7C). In addition to fitting the seven kinetic parameters in the equations, an eighth parameter was used to characterize the reduction in iron availability in the 100 μM DTPA condition (Table 1). We set [*Fe*] = 1 in the absence of DTPA and [*Fe*] = 𝑝*_DTPA_* in the presence of 100 μM DTPA, in dimensionless units of fraction of [Fe] in M9 + lactose (changes in iron levels during the experiment were presumed negligible). The model accurately captured total population size (OD_600_) with great accuracy and successfully recapitulated the growth dynamics of *S. enterica* and *E. coli* populations within co-culture (Fig. 6B, C, D). The exception was that the model predicted a sharp decline of wildtype *E. coli* cell numbers after 8 hours of co-culture with wildtype *S. enterica* SL1344 (Fig. 6B). However, 10^6^–10^7^ viable *E. coli* cells were always detected after 24 hours of co-culture (Fig. 1, S2). This maintenance of *E. coli* population could be a result of evolution of resistance, which was not incorporated into the model structure.

### *E. coli* subpopulations emerge in the strong selective pressures of co-culture

Considering the potency of ColIb, we hypothesized that the *E. coli* populations present at 24 hours are not wildtype. This was experimentally addressed by quantifying the proportion of ColIb-resistant *E. coli* in co-culture compared to *E. coli* monocultures. At 0 and 6 hours in co-culture and monoculture, the frequency of ColIb-resistant *E. coli* is very low, ranging from <0.0007% to 0.0049% (Fig. 6E). By 24 h, over 90% of members of an *E. coli* population in co-culture were resistant to ColIb, suggesting strong selection and outgrowth of a colicin-resistant subpopulation by 24 h. The elevated amounts of β-galactosidase measured in culture supernatants at 6 hours corresponded to a time when over 99.99% of *E. coli* were colicin sensitive (Fig. 3B and6E). PCR identified the acquisition of the *cib/imm* locus by some *E. coli* isolates, a known consequence of pCol1B9 conjugation in *S. enterica* and *E. coli* co-cultures [9, 10]. Whole-genome sequencing of a resistant clone that lacked *cib/imm* revealed a frameshift mutation that dramatically shortens the *cirA* gene product, CirA, from 663 amino acids to 269 amino acids, which we called *cirA*^tr^.

## Discussion

Cooperative co-culture of *E. coli* and *S. enterica* in lactose minimal media have been used extensively to understand fundamental properties of mutualism [25, 44, 45]. In the mutualist system, *E. coli* secretes acetate from metabolized lactose; in return, *S. enterica* LT2 secretes methionine [44]. Two recent studies are particularly pertinent to our findings. In one, researchers observed that *E. coli* grew poorly on cellular debris from lysis of *E. coli* or *S. enterica* in lactose minimal medium, presumably limited by the absence of active methionine production. *S. enterica* grew poorly when fed lysed *S. enterica* —presumably due to the inaccessibility of lactose— but grew 100-fold better when fed lysed *E. coli* [45]. A growth advantage dependent on cellular debris from *E. coli* is consistent with cross-feeding on released β-galactosidase.

Mutualist cross-feeding between *E. coli* and *S. enterica* in a lactose medium can be enhanced by evolution of galactose secretion by *E. coli*, though this is a costly form of cooperation [44]. At the outset of co-culture experiments in minimal medium with lactose, *E. coli* secretes low-value carbon sources like acetate. After 280 generations, *E. coli* subtypes that stopped metabolizing galactose repeatedly evolved through loss of *galK* function; these *galK*-deficient cells exported galactose that improved the growth of methionine-producing *S. enterica* [44]. Even though this prior evolutionary study demonstrated the benefit to *S. enterica* of galactose cross-feeding in the presence of other metabolites, the long time required to evolve the *E. coli* phenotype contrasts with the immediate access to galactose by community members from colicin-mediated release of β-galactosidase.

Bacteriocins have numerous documented impacts on microbial communities: they can benefit producers by interfering with competitors [11, 46–49], they can cause diversification within bacterial communities [50, 51], and bacteriocins can facilitate niche colonization [52–54]. *S. enterica* cells release colicin after dying [55, 56], precluding *S. enterica* cells from being both producers and beneficiaries. Thus, the colicin-mediated interactions we describe here manifest only for interacting populations, not on the level of single cells. The discovery of posthumous release of active β-galactosidase by *E. coli* presents a mechanism through which cells contribute to community interactions after death. In this case, extracellular β-galactosidase becomes a public good that improves nutrient availability for the community members that can evade or resist the colicin.

Resistance to colicins can occur through: alteration of membrane transporters [57]; the addition of siderophores to culture media to block *cirA* prior to colicin treatment [36]; plasmid and immunity gene transfer to *E. coli* in co-culture with *S. enterica* [9]; and spontaneous mutations in transporters or their regulators [35]. We observed mutations in the ColIb receptor *cirA* in *E. coli* as well as transfer of the colicin and immunity *cib*/*imm* genes to *E. coli*. In our co-culture experiments, *E. coli* populations were colicin-resistant by 24 h, which highlights the rapid rate that a small (approximately 1 in 5000 cells), insensitive subpopulation can grow to dominance. It remains unclear what proportion of resistance in replicate co-cultures or even within an *E. coli* population is attributable to *cirA* mutation or to acquisition of *imm*. Each of the resistance mechanisms originates in single *E. coli* cell in a population of completely inhibited *E. coli*, which allows a resistant clone to expand through many generations to become the dominant *E. coli* variant in the flask. We have yet to quantify interactions in a structured environment where physical constraint of clonal expansion is predicted to limit the growth of resistant subpopulations. Loss of *cirA* function is expected to impart a high cost because *cirA* is central to iron uptake. Therefore, *cirA*^tr^ mutants are not predicted to persist in nature even if such a mutation is initially selected in the presence of a colicin-producing community member. Further experimentation on the rates at which resistance develops is important for current efforts to apply bacteriocins as antimicrobials in food and health applications [58].

Dynamic mathematical models can provide insights for community stability, alternative stable states, and community succession [59]. In our study, the ordinary differential equation model was developed from a larger model by reducing features that were identified as negligible or that could not be well-characterized. The reduced model successfully predicted experimental community behaviours, confirming that modeled parameters represent salient ecological features that dictate community dynamics in our synthetic community. Although successful in producing a suitable fit, this preliminary model could be improved in several ways. In particular, we highlight the fact that the model’s description of iron dependence is calibrated against just two distinct media iron conditions, limiting confidence in model-based extrapolations to other iron levels. In addition, the model’s description of populations in terms of OD_600_ allows a consistent treatment of the available data but means that the model cannot be used to make predictions of viable cell counts (e.g. CFUs).

The strong upregulation of galactose transport demonstrated the importance of the new sugar source to cells (Fig. 3). Additionally, *S. enterica*’s improved growth in spent media from colicin-containing co-cultures demonstrated a benefit of released β-galactosidase activity (Fig. 4). However, a limitation of our study was that we were unable to establish conditions where cross-feeding occurred exclusively through the monosaccharides produced by β-galactosidase activity. Instead, extracellular β-galactosidase activity always occurred in the presence of overflow metabolites like acetate. The rich medium (LB) experiments (Fig. 4C) benefited from robust growth of co-cultures to make spent media. However, extracellular β-galactosidase activity added to a residual pool of carbon and energy sources. How might cross-feeding from lytic release of enzymes benefit toxin producers in nature? Even small growth benefits detected in laboratory conditions can reflect significant fitness gains, and we are using the sensitivity of competitive growth assays to measure fitness of mutants that are unable or less able to access glucose and galactose in co-culture [60].

Researchers have previously identified export of glucose and galactosides in vitro by the *E. coli* the sugar efflux transporter SetA [61], but *set* genes are poorly expressed in laboratory conditions [62] and in vivo experiments did not observe efflux by SetA [63]. In the present study, setA and other genes in the SgrR glucose phosphate stress regulon were not transcriptionally changed in the RNA-seq data. Thus, there is no reason to suspect *E. coli* is actively secreting galactose in response to colicin killing.

Colicin-mediated antagonism between *E. coli* and *S. enterica* has been studied in the context of competition for iron in mouse intestines and laboratory conditions [9, 10]. Our findings suggest that cross-feeding in mammalian intestines can occur both through overflow metabolism from intact *E. coli* or from colicin-induced lytic release of β-galactosidase activity, even in the absence of inflammation and competition for iron. Intestinal microflora may benefit from *E. coli* catabolism of lactose in juvenile mammalian intestines [64]. Less familiar is the ability of β-galactosidase to degrade plant-derived galactolipids [65], raising the possibility β-galactosidase cross-feeding could benefit colicin producers by degrading plant materials in mammalian intestines. *E. coli* and *S. enterica* co-occur in a wide diversity of habitats, including plant-associated niches, freshwater, and terrestrial environments, raising the intriguing possibility that colicin-mediated cross-feeding occurs in a wide range of ecosystems where a lytic toxin can amplify cross-feeding potential.

## Supplementary Material

SUPPLEMENTARY_combined_Lerminiaux_et_al_wraf032

## Data Availability

The datasets generated during and analysed during the current study are available in the Sequencing Read Archive, https://www.ncbi.nlm.nih.gov/sra/PRJNA786616. Code for the mathematical model is available at GitHub, https://github.com/ingallslab/colicin-ODE-model/tree/main.
