# Supplementary material for: Lysis of Escherichia coli by colicin Ib contributes to bacterial cross-feeding by releasing active β-galactosidase
Source: ISME J. 2025 Feb 19;19(1):wraf032. doi: 10.1093/ismejo/wraf032 (PMC11896792; doi:10.1093/ismejo/wraf032)
Supplement: SUPPLEMENTARY_combined_Lerminiaux_et_al_wraf032 [file supplementary_combined_lerminiaux_et_al_wraf032.pdf]

Supplementary Figures

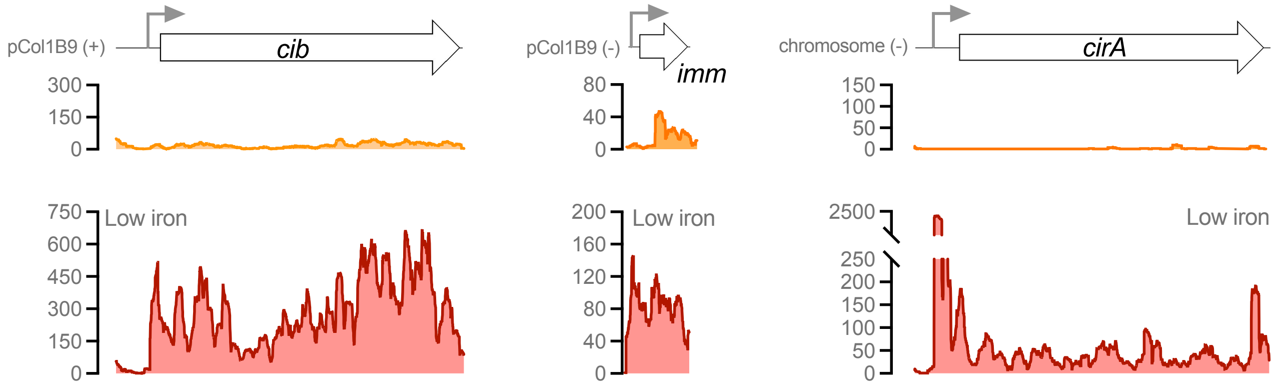

Figure S1. Changes in transcript abundances measured in [1] was used to examine expression of *cib, imm,* and *cirA* in *S. enterica* Typhimurium ST4/74 growing in L-broth during mid-exponential phase (top, orange) and following low iron shock (bottom, red).

The ferric uptake regulator (Fur) protein represses *Salmonella cib* and *imm* expression in the presence of iron, and similarly represses transcription of the *cirA* iron receptor gene in *E. coli* (**Fig. 1**). Hence, production of ColIb is coordinated with the availability of its specific receptor, CirA. Examining the transcription of *cib*, *imm*, and *cirA* in a previously published *S. enterica* Typhimurium RNA-seq dataset confirmed that the genes are strongly upregulated on transition to iron-limited conditions (above) [2]. In addition to direct transport of iron by envelope-bound transporters like CirA, *E. coli* and *S. enterica*. We measured *cib* expression in the *S. enterica* SL1344 *ΔentA* mutant, which cannot produce enterobactin or salmochelin. *cib* expression in mutant and wildtype was highest at 7 hr and declined in both strains by 24 hr (**Fig. S5**). Higher *cib* expression at 24 hr in the *ΔentA* mutant compared to wildtype suggests that iron starvation triggered Fur activity in *ΔentA* mutant cells. We next tested if ColIb killing of *E. coli* causes downregulation of siderophore production in *S. enterica*, as predicted if iron is liberated from *E. coli* and competition is reduced by ColIb killing.

[1] Canals R, Hammarlöf DL, Kröger C, Owen SV, Fong WY, Lacharme-Lora L, et al. Adding function to the genome of African Salmonella Typhimurium ST313 strain D23580. *PLOS Biology* 2019; **17**: 3000059.

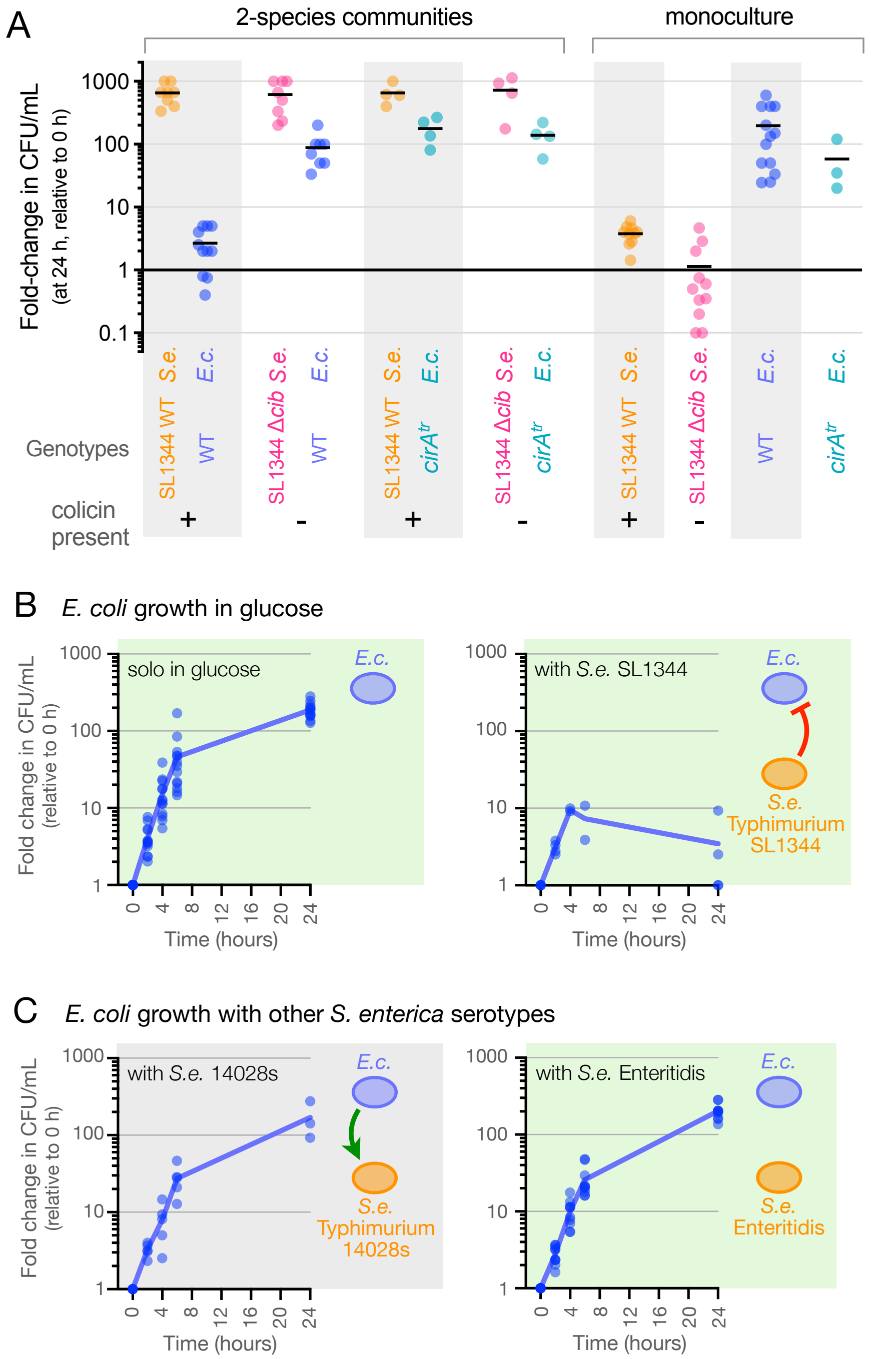

Figure S2. Colicin produced by *S. enterica* (*S.e.*) SL1344 inhibits *E. coli* (*E.c.*) growth. (A) Fold-change in abundance after 24 h for co-cultures and monocultures. Cultures were started at 1x10^{6} CFU/mL in M9+lactose and grown in a plate reader for 24 h at 37 ∘C shaking at 200 rpm. Horizontal bars represent means of 3 to 11 biological replicates. We note that the *E. coli cirA*^{tr} mutant was isolated by selection for colicin resistance, described in **Supplementary Table 1**. **(B)** Growth curves plotting fold-change in *E. coli* cell numbers over 24 h in monoculture or co-culture with *S.e.* Typhimurium SL1344 in flasks. **(C)** Growth curves plotting fold-change in *E. coli* cell numbers over 24 h in co-culture with *S.e.* Typhimurium 14028s in lactose or with *S.e.* Enteritidis in glucose in flasks. Each species was started at 1x10^{7} CFU/mL in M9+lactose (grey shading) or M9+glucose (green shading).

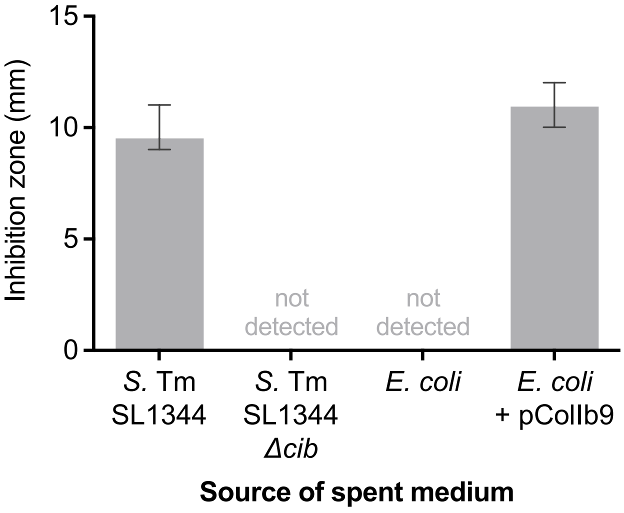

Figure S3. Inhibition of *E. coli* growth by cell-free spent medium. Spot assay with spent medium (cell-free supernatant) from *S. enterica* or *E. coli* cultures. Cell-free medium from *S. enterica* SL1344 wildtype contains ColIb that inhibits growth when spotted on a lawn of wildtype *E. coli* (**Fig. 2D** in main text). Deletion of the gene *cib* in *S. enterica* SL1344 removes all detectable inhibition of *E. coli* growth. Conversely, transfer of pColIb9 to *E. coli* confers colicin production, as demonstrated by a strong zone of inhibition when spent media is spotted on wildtype *E. coli*. Each spent medium was filtered and spotted twice (technical replicates). Mean and ranges of 4 to 8 biological replicates are plotted.

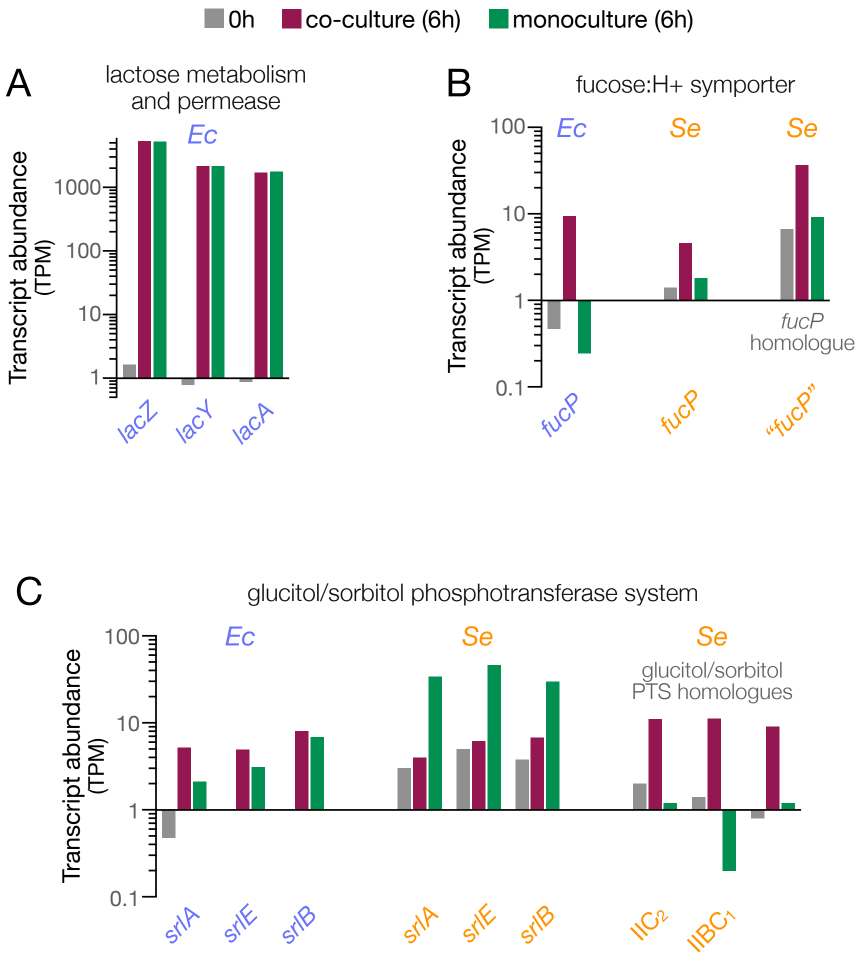

Figure S4. Monosaccharide uptake gene expression in co-cultures and monocultures. (A) Transcript abundances of *E. coli* lactose metabolism and uptake genes *lacZYA*. **(B)** Transcript abundances of monosaccharide uptake genes. The *fucP* gene, which encodes a fucose:H+ symporter, was the most differentially upregulated in *E. coli* co-culture (**Fig. 3C** in main text). *S. enterica* encodes two genes annotated as *fucP*, one 92% and the other 35% identical to the *E. coli* FucP homolog at the amino acid level. Both of the *S. enterica fucP* genes were more active in co-culture. Fucose is a monosaccharide component of lipopolysaccharide [2], making it unclear why co-culture specifically triggers this symporter. **(C)** Transcript abundances of phosphotransferase monosaccharide uptake genes. *S. enterica* encodes two homologs of the glucitol/sorbitol phosphotransferase system encoded by *srlAEB* in *E. coli*. The more divergent homologous system is strongly induced in *S. enterica* by co-culture.

[2] Gunn FJ, Tate CG, Henderson PJF. Identification of a novel sugar-H^+ symport protein, FucP, for transport of L-fucose into *Escherichia coli*. *Molecular Microbiology* 1994; **12**: 799–809.

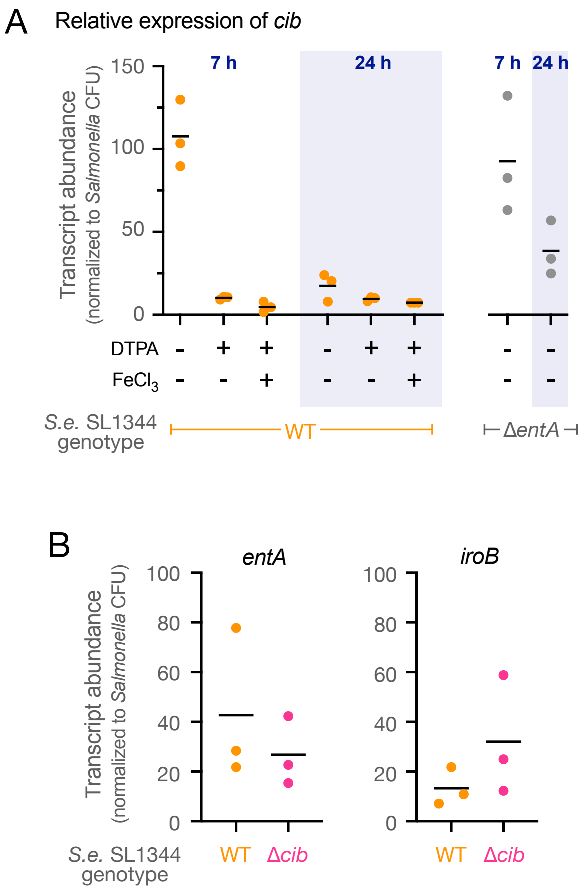

Figure S5. Colicin and siderophore gene expression. (**A**) Relative transcript quantity of colicin gene *cib* in *Salmonella* in different co-culture conditions. *S. e.* WT and *E. coli,* and *S. e.* Δ*entA* and *E. coli* co-cultures were started at $1x10^6$ CFU/mL in M9+lactose and RNA samples were taken at 7 h and 24 h. Several cultures had 100 µM DTPA and 16 µM $FeCl_3$ added at 0 h. Bars represent the mean and each point represents a biological replicate. A two-way ANOVA was performed to analyze the expression of *cib* in *S.e.* WT at different time points and culture conditions (M9+lactose, +DTPA, and +DTPA +$FeCl_3$). Simple main effects analysis showed that culture condition (F (2, 12) = 37.20, p<0.0001), time point (F (1, 12) = 5.189, p=0.0418), and interaction between culture condition and time point (F (2, 12) = 14.81, p=0.0006) had statistically significant effects on gene expression. Šídák's test for multiple comparison found that the mean gene expression was significantly different between *S.e.* WT at 7 h and 24 h in M9+lactose (p_{adj} = 0.0003). No other comparisons were statistically significant. A second two-way ANOVA was performed to analyze the effect of time and strain (*S.e.* WT and *S.e.* Δ*entA*) on *cib* expression in M9+lactose. Simple main effects analysis showed that time (F (1, 8) = 33.35, p=0.0004) had statistically significant effects on gene expression, though strain and the interaction between time and strain did not. Šídák's test for multiple comparisons found that no comparisons were statistically significant. (**B**) Relative transcript abundance of siderophore genes *entA* and *iroB* in *S. e.* WT and *S. e.* Δ*cib* when co-cultured with *E. coli* WT. Co-cultures were started at $1x10^6$ CFU/mL in M9+lactose and RNA samples were taken at 0 h and 7 h. qPCR quantification is the result of averaging two technical replicates and *cib* transcript values were taken from a standard curve and normalized to transcripts at 0 h. A two-way ANOVA was performed to analyze the expression of each gene in co-culture with and without colicin. Simple main effects analysis showed that culture condition (colicin +/-) did not have a statistically significant effect on gene expression (F (1, 8) = 0.2852, p=0.6078).

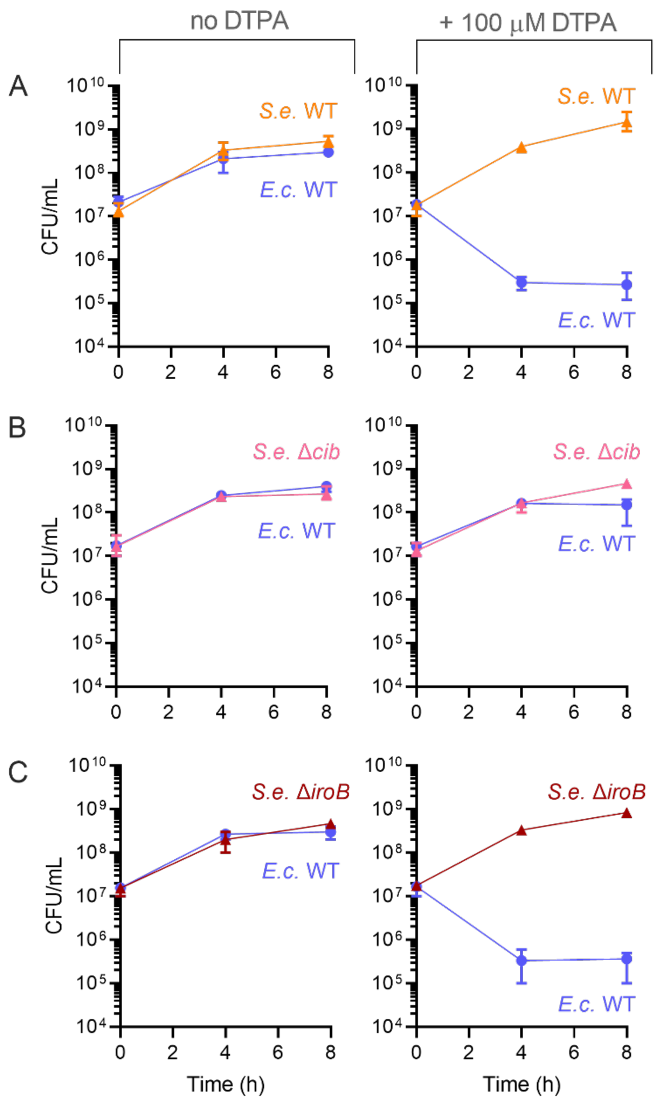

Figure S6. DTPA-induced colicin killing. (**A**) *S.e.* WT and *E.c.* WT, (**B**) *S.e. Δcib* and *E.c.* WT, and (**C**) *S.e. ΔiroB* and *E.c.* WT were grown for 8 hours in nutrient-rich LB. 100 μM DTPA was added to some cultures to induce iron starvation and colicin production. Each point is the average of three biological replicates and error bars indicate range.

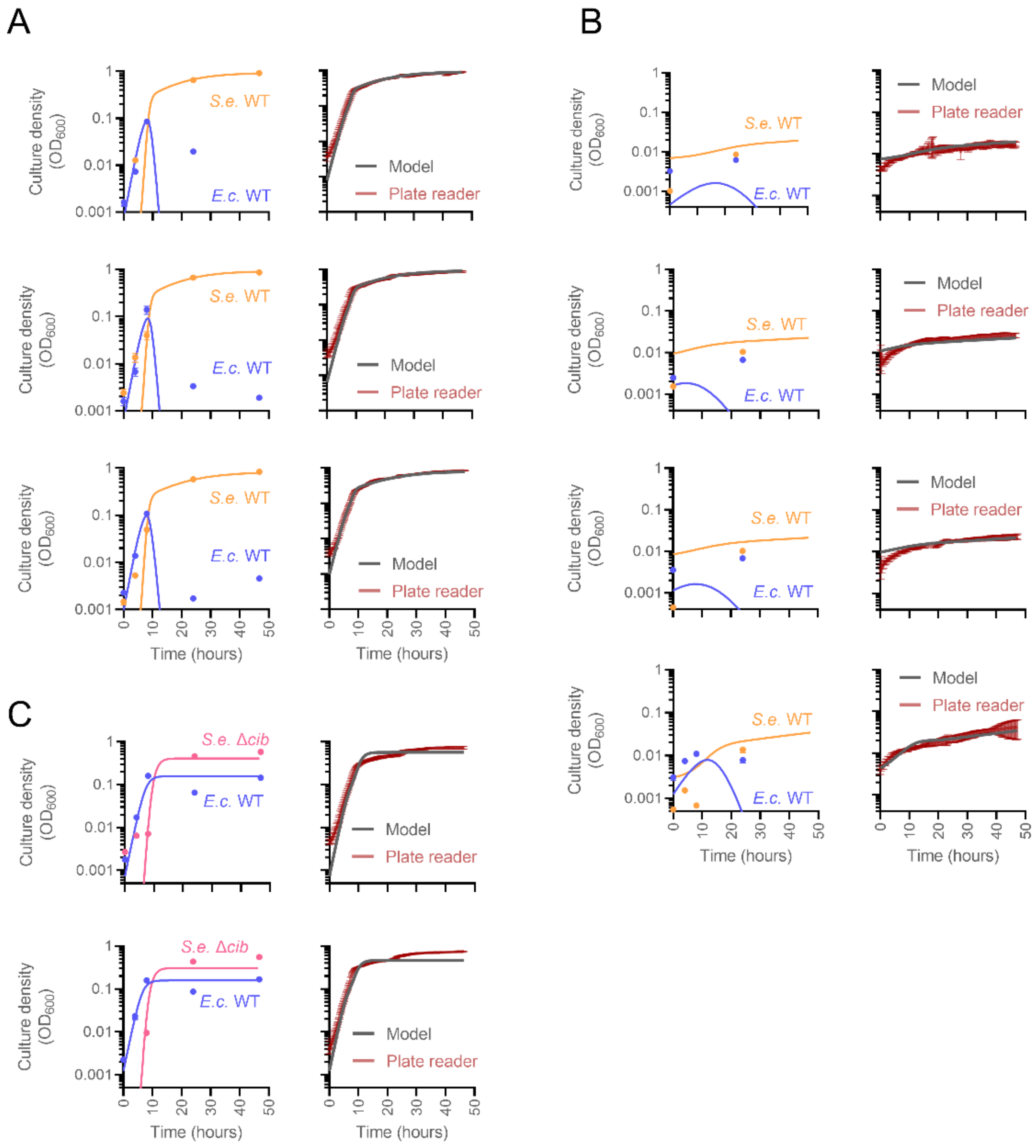

Fig. S7. Model calibration against data from three experimental scenarios. Representatives from each scenario are plotted in **Figure 6**. Lines represent model output for each species and total biomass, and points represent experimentally measured biomass. (A) co-culture of *S.e.* SL1344 WT and *E.c.* WT in M9+lactose, (B) co-culture of *S.e.* SL1344 WT and *E.c.* WT in M9+lactose with 100 μM DTPA, and (C) co-culture of *S.e.* SL1344 *Δcib* and *E.c.* WT in M9+lactose. Plate reader measurements (red) are plotted as mean and standard deviations of three or more biological replicates.

Supplementary Methods

Bacterial strains

Strains and plasmids used in this study are listed in **Supplementary Table 1.** Siderophore mutants (Δ*iroB* and Δ*entA*) were generated by transducing mutants from Porwollik et al., 2014 into wild-type SL1344 or 14028 with P22 HT 105 bacteriophage. Mutants were verified with PCR. The source Δ*iroB* and Δ*entA* mutant strains were supplied by BEI Resource (www.beiresources.org).

E. coli W3110 contained plasmid pOT1 to enable gentamicin selection and is referred to as '*E. coli* WT' for simplicity in the main text. A point mutation in *hisG* was selected in *S. enterica* SL1344 to restore ancestral histidine synthesis; this SL1344 derivative is referred to as '*S. enterica* SL1344 WT' for simplicity in the main text. Culture media compositions are detailed in supplementary methods. *E. coli* strains were experimentally confirmed to not grow in the presence of streptomycin, chloramphenicol, or kanamycin; *S. enterica* strains were experimentally confirmed to not grow in the presence of gentamicin.

Generation of colicin mutant (Δ*cib*) by allelic exchange

To generate a *S. enterica* pCol1B9 plasmid without the colicin and immunity genes, the colicin gene *cib* and adjacent immunity gene *imm* were replaced with a kanamycin resistance marker. Mating experiments between SL1344 WT and S17-1λpir pM1436 were performed as previously described (Stecher et al., 2012) and were diluted 1/10 on agar selective for SL1344 transconjugants (M9 glucose + 1.5% agar + 20 µg/mL kanamycin) for 48 h. Transconjugatants were toothpicked onto a lawn of *E. coli* W3110 WT and screened for colicin production; colonies that had little/no zone of inhibition due to a mutant *cib* were inoculated into LB + 80 µg/mL kanamycin. DNA was extracted with the EZ-10 Spin Column Genomic DNA kit (BioBasic) and mutants were evaluated by PCR with p2-cibimm/flank-F 5'-ACTGTATTCCGGGAGATTCG-3' p2-cibimm/flank-R 5'-GAGAGTGCTGATTGTGCTGG-3'. As pCol1B9 is a multi-copy plasmid, colonies were inoculated into LB + 600 µg/mL kanamycin to ensure all plasmid copies lost *cib* and *imm*. Mutants were confirmed by PCR and by phenotypic loss of the zone of colicin inhibition in spot assays with *E. coli* W3110 WT.

Supplementary Table 1. Bacterial strains and plasmids

Species and Strain	Relevant properties	Reference
E. coli		
W3110	pOT1, gentamicin^R	ATCC 39936; Allaway et al., 2001
+ 32/93	W3110 *cirA*^{tr}; isolated from spent media treatment	This work
S17-1	λpir	Simon et al., 1983
S. enterica sv. Typhimurium		
SL1344 WT	*his*$^+$, streptomycin^R	Hoiseth and Stocker, 1981
Δ*iroB*	SL1344 Δ*iroB*, kanamycin^R	This work; Porwollik et al., 2014
Δ*entA*	SL1344 Δ*entA,* chloramphenicol^R	This work; Porwollik et al., 2014
Δ*cib*	SL1344 Δ*cib*Δ*imm*, kanamycin^R	This work; Stecher et al., 2012
14028 WT		ATCC 14028
Δ*iroB*	14028 Δ*iroB*, kanamycin^R	This work; Porwollik et al., 2014
Δ*entA*	14028 Δ*entA,* chloramphenicol^R	This work; Porwollik et al., 2014
Plasmids		
pM1436	Kanamycin^R	Stecher et al., 2012
pOT1	Gentamicin^R	Allaway et al., 2001

Culture conditions

All growth media was made with autoclaved Milli-Q water. Luria Bertani (LB) broth or LB agar (1.5%) contained 0.5% NaCl. M9 minimal medium (1X M9 salts [33.7 mM Na2HPO4, 22.0 mM KH2PO4, 8.55 mM NaCl, 9.35 mM NH4Cl], 1 mM $MgSO_4$, 0.1 mM $CaCl_2$) contained one of the following carbon sources: lactose (4 mM), glucose (4 mM), or glycerol (4 mM). Where applicable, antibiotics were supplemented at the following concentrations: gentamycin (20 µg/mL), streptomycin (50 µg/mL), chloramphenicol (30 µg/mL) and kanamycin (25 µg/mL). Diethylenetriaminepentaacetic acid (DTPA) crystals were suspended in water to a stock concentration of 10 mM (pH = 7.13). Final concentrations of DTPA in culture medium are specified in the text. $FeCl_3$ solution was made fresh and used the same day in cultures containing DTPA to restore iron availability. Because the oxidation state and solubility of iron is impacted by pH, salt, and other components of the culture medium, not

all iron will be bioavailable upon supplementation. Thus, although the absolute concentration is difficult to determine, restored bacterial growth will reflect bioavailable iron in the culture medium.

Polymicrobial culturing

All bacterial cultures described in this work were started fresh from a single colony streak isolated from -80 °C freezer cultures. Starting each experimental culture from a single colony ensured clonal populations and greatly reduced the potential for contamination of cultures. To start experimental cultures, each strain was pre-grown overnight at 37 °C with 200 rpm shaking in M9+lactose (*E. coli*) or M9+glucose (*S. enterica*) inoculated with a single colony. In the morning, cells were subcultured into fresh M9 medium with a starting OD_{600} = 0.075, followed by incubation at 37 °C with 200 rpm shaking for 2-3 h to reach OD_{600} = 0.1 - 0.2. Cultures were then passed through a 0.2 µm filter funnel with a vacuum to remove the supernatant and washed with pre-warmed carbon-free M9 medium. The filters were transferred to flasks containing 10 mL fresh pre-warmed M9+lactose medium and were incubated at 37 °C with 200 rpm shaking for 15 mins to dislodge cells from the filters. For co-culture experiments in flasks, cultures were combined to a starting OD_{600} = 0.05.

For co-culture experiments in the plate reader, cultures were combined to a starting OD_{600} = 0.0075 (about $1x10^6$ CFU/mL) unless stated otherwise, and this initial OD_{600} reading was determined with a Genesys 20 spectophotometer (Thermo Scientific, MA, USA). Optical density experiments were performed at 600 nm (OD_{600}) in the SynergyHT Microplate Reader (BioTek, VT, USA). Reads were taken every 15 minutes over a minimum of 24 h and cells were incubated at 37 °C with constant shaking. 50 μL of light mineral oil was overlaid on 250 μL of culture in each well to prevent desiccation.

CFUs were determined by 10 μL drop plates from a 10^{-2} to 10^{-10} ten-fold dilution series, using plates with streptomycin (*S. enterica* SL1344 WT), kanamycin (*S. enterica* SL1344 *Δ*, *S. enterica* 14028 WT), chloramphenicol (*A. baumannii*), or gentamicin (*E. coli* WT). *E. coli* W3110 contained plasmid pOT1 to enable gentamicin selection. *E. coli* cannot grow in the presence of streptomycin, chloramphenicol, or kanamycin

which was experimentally confirmed; *S. enterica* cannot grow in the presence of gentamicin or chloramphenicol which was experimentally confirmed.

Co-culture RNA-seq

Cultures were pre-grown in M9+glucose (*E. coli*) and M9+glycerol (*S. enterica*) as above, were vacuum-filtered and washed with 15 mL of M9+lactose to remove residual carbon sources. Filters were transferred into pre-warmed M9+lactose to achieve a starting density of $1x10^7$ CFU/mL. Cultures were sampled at 0 h and 6 h in both co-culture and monoculture (schematic in main text **Fig. 2A**).

Cells were fixed in RNA*later* (Invitrogen, CA, USA) according to manufacturer guidelines and pellets were frozen. RNA was extracted from thawed pellets using RNeasy spin column extraction with on-column DNase treatment, as per manufacturer instructions (Qiagen). The Illumina Stranded Total RNA Prep, Ligation with Ribo-ZeroTM Plus (Illumina, CA, USA) was used to conduct ribosomal RNA depletion and sequencing library synthesis with random hexamer primed reverse transcription. Samples were sequenced by paired-end sequencing on the Illumina NextSeq, which generated over 20 million reads per sample.

FastQC (Andrews, 2010) was used to assess read quality and Trimmomatic v0.35 (Bolger et al., 2014) was used to remove adapter sequences and eliminate reads with an average Qscore < 20 across a 4-bp sliding window. RNA-seq analysis was computed with the READemption v0.5.0 pipeline (Förstner et al., 2014), using default parameters except for the align step, where we included `--crossalign_cleaning` argument to eliminate reads that mapped equally well to both species. Coverage graphs were normalized by the total number of aligned reads and then multiplied by the lowest number of aligned reads of all considered libraries (coverage-tnoar_min_normalized). Transcript per million (TPMs) calculated by READemption were used to compute fold-change differences in gene expression for the three-species co-culture.

Reverse transcriptase quantitative PCR (qPCR)

Cells were fixed in ice-cold 5% phenol/95% ethanol for 2 h, and RNA was isolated using the EZ-10 Spin Column total RNA kit (BioBasic). DNase treatment was done for each sample using the TURBO DNA-free® kit (Ambion),

and cDNA template was synthesized by random priming using the Verso cDNA kit (Thermo Scientific). qPCR reactions were conducted in duplicate for each primer set using IQ™ SYBR® Green Supermix (Biorad) for 40 cycles. The oligonucleotide primers used are found in **Supplementary Table 2**. To test for primer dimers, melt curves were performed for all primer sets and no template control reactions were also conducted. Standard curves were produced for each primer using five serial 10-fold dilutions of chromosomal DNA as template. Gene expression was normalized to the ratio of *S. enterica* in co-culture as determined by CFU counts, or to expression at 0h in *S. enterica* monoculture.

Supplementary Table 2. qPCR primer sequence information for colicin and related genes

**Target**	**Primer**	**Sequence (5'-3')**	**Average R-squared**	**Reference**
cib	cibF<br>cibR	TGAGTGGTGATCCGAGATTTG<br>CCGGAATGGGTCAGAAGATAAT	0.999	This work
cirA	cirAFor<br>cirARev	GTTACCGTCGATACCACCATTC<br>TTCCCAGCACACCATCAATTA	0.999	This work
mglB	mglBF<br>mglBR	CGTGATGGCAAGTCTGTTATTC<br>ACGCACCACGGACATAAA	0.999	This work
galP	galP_F<br>galP_R	TGCAGGTTAAACAGAGCGGT<br>GCTGCATGACCTGCAACAAA	0.998	This work
entA	entAF<br>entAR	GGTTTCGGCGAACAGTTTAAG<br>CAGATCGGAGGCAAGAAACA	0.999	This work
iroB	iroBF<br>iroBR	CCACTGATTGCCGCTAAGTA<br>GTGATTTCGTCACGCCTTTG	0.997	This work

β-galactosidase assay

Cultures were grown in M9+lactose as described above and at 7 h, the supernatants were sampled for β-galactosidase using the Beta-Glo Assay System (Promega, WI, USA). Cultures were filter-sterilized through a 0.2 µm filter, and 100 µL of supernatant was added to 100 µL of reagent in a white-walled 96-well plate in triplicate. The plate was mixed and incubated in the dark for 30 mins at room temperature. Luminescence measurements were made with the SynergyHT Microplate Reader (BioTek, VT, USA), with an integration time of 0.5 s and gain set to 100. Background luminescence was subtracted from the readings and relative light units were normalized to the luminescence from the supernatant of *E. coli* monocultures.

Preparation of cell-free spent media and colicin resistance assay

A colicin overlay on agar plates was used to calculate the percentage of *E. coli* that became resistant to colicin. To prepare cell-free medium containing colicins, stationary *S. enterica* SL1344 WT cultured in LB for 24 h was centrifuged at 8,000 x g for 10 minutes to pellet cells then the resulting supernatant was filter sterilized with a 0.2 µm syringe filter. For colicin+ LB agar plates, 200 µL of colicin-containing spent media was spread using glass beads and the plates were allowed to dry for 60 minutes before being used. Two additional cultures were started 6 hours and 24 hours after the first and processed in the same manner to have fresh spent media for each time point. For colicin-negative plates, 200 µL of plain LB was spread using glass beads and the plates were allowed to dry for 60 minutes before being used.

Monocultures and co-cultures began with inoculating 25 mL pre-warmed M9+lactose in flasks with 0.0625 OD units of *E. coli* WT or 0.0625 OD units of each of *S. enterica* SL1344 WT and *E. coli* WT, respectively. Immediately after inoculation, after 6 hours of growth, and after 24 hours of growth, samples were removed, diluted in M9 + lactose, and 50 µL was spread plated on LB agar +/- colicins supplemented with 20 µg/mL gentamicin in triplicate. CFU/mL for each timepoint was calculated from manual colony counts and dilution factors.

Whole genome sequencing and library preparation

DNA was extracted with the EZ-10 Spin Column Genomic DNA kit (BioBasic) and sheared for library preparation by Bioruptor® Plus (Diagenode) using four low intensity cycles of 10s on / 90s off at 4 °C. DNA libraries were prepared for sequencing using NEBNext® Ultra™ II DNA Library Prep Kit for Illumina according to manufacturer’s instructions. Isolates were sequenced by paired-end sequencing on the Illumina® MiSeq® using MiSeq® Reagent Kit V2 300 cycle kit. The kit allows up to 15 M total reads at 2 x 150 bp read length and we obtained 64-fold coverage on each genome.

Spot assays of colicin activity

Overnight *E. coli* WT cultures were diluted to McFarland turbidity standard = 2.0 (OD_{600} = 0.242) and 300 µL of the cell culture was spread on LB agar containing gentamicin. 10 µL of filter-sterilized spent medium from stationary *S. enterica* SL1344 WT in LB were dropped on the inoculated plates in triplicate and plates were incubated at 37°C for 24 h.

β-galactosidase cross-feeding assay

E. coli and *S. enterica* strains were grown to early stationary phase in shaking flasks (250 RPM) in LB (half sodium; 5.0 g/L NaCl), 100 µl of 10 mM DTPA, and 100 µl of 0.8 M IPTG. Stationary *E. coli* and either *S. enterica* 1344 WT or *S. enterica* SL1344 Δ*cib* were added in a 1:1 ratio to a final volume of 25 ml then co-cultured for 4 hours with shaking at 37 ºC. No fresh LB was added, but 50 µl of 10 mM DTPA and 50 µl of 0.8 M IPTG was added. Co-cultures were filter sterilized using 0.2 µm filter cups (Thermo Scientific) into sterilized vacuum flasks. Filtered media was plated on LB agar plates to ensure sterility. Five mL of spent media were inoculated in tubes with stationary *S. enterica* SL1344 WT that was washed by centrifugation and resuspension in spent media. Where specified in the text, lactose (0.275 mM) or glucose (0.55 mM) were added to spent media immediately before addition of cells. Tubes were angled at 45 degree and shaken at 250 RPM at 37 ºC.

Statistical analyses

All data was log-transformed prior to analysis to approximate normal (Gaussian) distribution. Statistical analyses were performed with GraphPad Prism version 9.3.1. Depending on the analysis, the one-way analysis of variance (ANOVA) test was used, or two-way ANOVA test followed by either Tukey's test for multiple comparisons (comparing all pairwise interactions) or Šídák's test for multiple comparisons (comparing independent groups). P values less than 0.05 were considered significant.

Model Calibration

Model simulations were generated with the ode23s numerical integration routine in MATLAB (MathWorks, MA, USA). All code is posted here [github link]. As described above, the calibration data (training data)

consisted of duplicate or triplicate observations of total population counts (inferred from OD600 plate reader measurements) and relative population size (from plate counts). Data were collected from three experimental scenarios (co-culture of wildtype *E. coli* and *S. enterica* SL1344 in M9+lac; co-culture of wildtype *E. coli* and *S. enterica* SL1344 in M9+lactose with 100 µM DTPA; co-culture of colicin-mutant *S. enterica* SL1344 Δ*cib* and wildtype *E. coli* in M9+lac; **Fig. 8** and **Fig. S3**). These data were used to calibrate the seven model kinetic parameters along with an eighth parameter, p_{DTPA} that characterizes the reduction in iron availability in the 100 µM DTPA condition (**Table 1**). The addition of 100 µM DTPA was simulated by setting [*Fe*] = p_{DTPA}. The Δ*cib* mutant was assumed to be insensitive to colicin; its behaviour is simulated by setting the colicin-induced death rate k_c = 0. For each parameterization $\mathbf{p}$, model predictions were compared against the training data via the weighted sum of squares function:

$$SSE(p) = \sum_i \sum_j \frac{1}{N_{i,j}} \sum_k \frac{\left(y_{obs}^{i,j}(t_k) - y_{sim}^{i,j}(t_k, p)\right)^2}{\max\left(\left(\sigma^{i,j}(t_k)\right), 0.001, (0.01) y_{obs}^{i,j}(t_k)\right)}$$

Where $y_{obs}^{i,j}(t_k)$ is the mean of the replicate measurements of observable *j* in experiment i at time t_k, $\sigma^{i,j}(t_k)$ is the standard deviation of those duplicate or triplicate measurements, and $y_{sim}^{i,j}(t_k,)$ is the corresponding model prediction. Index *i* runs over the twelve training experiments (four with wildtype in M9+lac, five with wildtype in M9+lac+100 μM DTPA, three with mutant in M9+lac). Index *j* runs over the three observables (total population, *E. coli* fraction, *S. enterica* fraction), and *k* runs over the time-points for the corresponding experiment and observable. These totals, denoted $N_{i,j}$, vary from experiment to experiment: wildtype in M9+lactose had 5 timepoints for each population fraction and 188 timepoints for total population; wildtype in M9+lac+100 μM DTPA population fractions had 2 timepoints for 4 experiments, 4 timepoints for 1, total population had 188 timepoints for all five; mutant in M9+lactose had 5 timepoints for each population fraction and 186 timepoints for total population. Scaling by $N_{i,j}$ ensured that the data from the (few) plate count timepoints are not swamped by the data from the far more numerous plate count timepoints. The absolute

and relative threshold parameters $(0.001, (0.01)y_{obs}^{i,j}(t_k))$ were introduced to mitigate the effect of outliers. Finally, we note that the three observables (total population and two population fractions) are not independent. We chose to incorporate all three of the corresponding datasets directly to ensure a consistent treatment of the variability in measurements (as opposed to, e.g. considering a population ratio, for which variability would be nonlinearly transformed).

This SSE was minimized by application of global optimization routines (simulated annealing, MATLAB function simannealbnd, and interior point algorithm, MATLAB function fmincon), with multiple starting points to improve the chance of finding the global minimum. Parameter values were bound to the range [0, 100]. The initial values of the *E. coli* and *S. enterica* populations were treated as free parameters to avoid over-weighting the measurements at these time-points. The best-fit initial conditions are posted at https://github.com/ingallslab/colicin_model. For each simulation the initial abundance of lactose was set at 4 mM and the initial abundance of sugar monomer was set to 0 mM.

Model uncertainty analysis

Uncertainty analysis was applied to provide a measure of confidence in the best-fit estimates of the kinetic parameters. The best-fit initial conditions were excluded from this analysis, as they only convey information about the scenarios corresponding to the training data.

Sensitivity coefficients

Local absolute sensitivity coefficients were defined as:

$$S_{i,j}(t_k) = \left.\frac{\partial y_{\text{sim}}^i(p, t_k)}{\partial p_j}\right|_{t=t_k}$$

where $\partial y_{sim}^i(p, t_k)$ is the i-th model output at time-point t_k and p_j is the j-th parameter. These derivatives were approximated by finite differences of 1% in p_j. Relative sensitivity coefficients were reached by scaling:

$$\widetilde{S_{i,j}}(t_k) = \frac{p_j}{y_{\text{sim}}^i(p, t_k)} S_{i,j}(t_k)$$

These were used for uncertainty analysis as follows. Overall sensitivity measures reported in **Table 1** were determined as:

$$\widetilde{S}_j = \sqrt{\sum_k \sum_j \left(\widetilde{S_{i,j}}(t_k)\right)^2}$$

where i runs over all observations and experiments, and t_k runs over all corresponding time-points. This provides an overall measure of the degree to which each model parameter p_j influences the model outputs that align with the measured data.

Identifiability scores

The orthogonalization approach of (Yao et al., 2003) was applied to account for correlation among the parameters, resulting in an assessment of identifiability, as follows. A sensitivity coefficient matrix was constructed by arranging the relative sensitivity coefficients for each parameter column-wise:

$$\tilde{\mathbf{S}} = \begin{bmatrix} \tilde{S}_{1,1}(t_1) & \cdots & \tilde{S}_{1,n_p}(t_1) \\ \vdots & \ddots & \vdots \\ \tilde{S}_{1,1}(t_{n_1}) & \cdots & \tilde{S}_{1,n_p}(t_{n_1}) \\ \tilde{S}_{m,1}(t_1) & \cdots & \tilde{S}_{m,n_p}(t_1) \\ \vdots & \ddots & \vdots \\ \tilde{S}_{m,1}(t_{n_m}) & \cdots & \tilde{S}_{m,n_p}(t_{n_m}) \end{bmatrix}$$

where n_p is the number of parameters, the observables 1 to m run over experiments and measurements, each with n_m time-points. The column with the largest 2-norm (square root of sum of squared entries) is labeled $\mathbf{X}_1$. The corresponding parameter is judged the most identifiable, and its identifiability score is this maximal 2-norm. The columns of $\tilde{S}$ are then each projected onto $\mathbf{X}_1$, with the residuals collected in matrix $\mathbf{R}_2$

$$R_2 = \tilde{S} - X_1(X_1^T X_1)^{-1} X_1^T \tilde{S}$$

The column of $\mathbf{R}_2$ with the largest 2-norm corresponds to the next most identifiable parameter. The matrix $\mathbf{X}_1$ is then concatenated with the column of $\tilde{S}$ that corresponds to that parameter, to form matrix $\mathbf{X}_2$. The residuals of the projection of $\tilde{S}$ onto $\mathbf{X}_2$ are then used to form matrix $\mathbf{R}_3$, and the third most identifiable parameter is identified. This process is iterated to provide an identifiability score for each parameter.

Confidence Intervals

Following (Emery and Nenarokomov, 1998; Gadkar et al., 2005), we construct the Fisher Information Matrix:

$$\mathrm{FIM} = S^T W S$$

where S is the vectorized sensitivities and W is the inverse of the measurement covariance matrix. Then assuming the measurement errors are independent and normally distributed, a lower bound on the radius of the 95% confidence interval for parameter p_j is given by:

$$1.96\sqrt{(FIM^{-1})_{jj}}$$

Given the sample sizes available in this study (triplicate observations), we could not estimate the full measurement covariance matrix. The measurement variances (diagonal entries in W) were calculated for each observation. The off-diagonal covariances terms were set to zero. The relative estimate is reported as a percentage.

Data availability

Sequencing reads were deposited in the Sequencing Read Archive https://www.ncbi.nlm.nih.gov/sra/PRJNA786616. Code for the mathematical model is available at GitHub, https://github.com/ingallslab/colicin-ODE-model/tree/main.

Acknowledgements

We thank Naomi Wee, Dr. Olivier Cunrath, Ashton Sies, Dr. Dinah Tambalo, Dr. Chris Yost, Mackenzie Hladun, Dr. Keith MacKenzie, Skip Olshefsky, Natisha Thakkar, Dr. Tzu-Chiao Chao, Dr. Jay Hinton, Dr. Kirsten Palmier,

Dr. Rosie Redfield, Valentyna Akulova, and members of IMSS for their helpful feedback, discussions, and support. Special thanks to Dr. Bärbel Stecher's group and BEI Resources for mutant bacterial strains.

Funding:

The research was funded by the Natural Sciences and Engineering Research Council of Canada (NSERC) Discovery Grants to ADSC (RGPIN-435784-2013 and RGPIN-2019-07135) and BPI (RGPIN-2018-03826); NSERC USRA, CGS-M, and PGS-D awards to NAL; and NSERC USRA awards to JMK and DMS.

References

Abdul-Tehrani, H., Hudson, A.J., Chang, Y.-S., Timms, A.R., Hawkins, C., Williams, J.M., Harrison, P.M., Guest, J.R., and Andrews, S.C. (1999). Ferritin mutants of *Escherichia coli* are iron deficient and growth impaired, and *fur* mutants are iron deficient. Journal of Bacteriology *181*, 1415–1428.

Allaway, D., Schofield, N.A., Leonard, M.E., Gilardoni, L., Finan, T.M., and Poole, P.S. (2001). Use of differential fluorescence induction and optical trapping to isolate environmentally induced genes. Environmental Microbiology *3*, 397–406.

de Almeida, R.A., Burgess, D., Shema, R., Motlekar, N., Napper, A.D., Diamond, S.L., and Pitt, G.D. (2008). A *Saccharomyces cerevisiae* cell-based quantitative β-galactosidase assay compatible with robotic handling and high-throughput screening. Yeast *25*, 71–76.

Andrews, S. (2010). FastQC: A Quality Control Tool for High Throughput Sequence Data [http://www.bioinformatics.babraham.ac.uk/projects/fastqc/].

Baker, M., Negus, D., Raghunathan, D., Radford, P., Moore, C., Clark, G., Diggle, M., Tyson, J., Twycross, J., and Sockett, R.E. (2017). Measuring and modelling the response of *Klebsiella pneumoniae* KPC prey to *Bdellovibrio bacteriovorus* predation, in human serum and defined buffer. Sci Rep *7*, 8329.

Bastos, M. do C. de F., Coelho, M.L.V., and Santos, O.C. da S. 2015 (2005). Resistance to bacteriocins produced by Gram-positive bacteria. Microbiology *161*, 683–700.

Bhattacharya, A., Pak, H.T.-Y., and Bashey, F. (2018). Plastic responses to competition: Does bacteriocin production increase in the presence of nonself competitors? Ecology and Evolution *8*, 6880–6888.

Bolger, A.M., Lohse, M., and Usadel, B. (2014). Trimmomatic: a flexible trimmer for Illumina sequence data. Bioinformatics *30*, 2114–2120.

Canals, R., Hammarlöf, D.L., Kröger, C., Owen, S.V., Fong, W.Y., Lacharme-Lora, L., Zhu, X., Wenner, N., Carden, S.E., Honeycutt, J., et al. (2019). Adding function to the genome of African *Salmonella* Typhimurium ST313 strain D23580. PLOS Biology *17*, e3000059.

Cascales, E., Buchanan, S.K., Duché, D., Kleanthous, C., Lloubès, R., Postle, K., Riley, M.A., Slatin, S., and Cavard, D. (2007). Colicin biology. Microbiology and Molecular Biology Reviews *71*, 158–229.

Chao, L., and Levin, B.R. (1981). Structured habitats and the evolution of anticompetitor toxins in bacteria. Proc. Natl. Acad. Sci. USA *78*, 6324–6328.

Chehade, H., and Braun, V. (1988). Iron-regulated synthesis and uptake of colicin V. FEMS Microbiology Letters *52*, 177–181.

Chikindas, M.L., Weeks, R., Drider, D., Chistyakov, V.A., and Dicks, L. (2018). Functions and emerging applications of bacteriocins. Current Opinion in Biotechnology *49*, 23–28.

Coyte, K.Z., Schluter, J., and Foster, K.R. (2015). The ecology of the microbiome: Networks, competition, and stability. Science *350*, 663–666.

Czárán, T.L., Hoekstra, R.F., and Pagie, L. (2002). Chemical warfare between microbes promotes biodiversity. Proc. Natl. Acad. Sci. USA *99*, 786–790.

De Roy, K., Marzorati, M., Van den Abbeele, P., Van de Wiele, T., and Boon, N. (2014). Synthetic microbial ecosystems: an exciting tool to understand and apply microbial communities. Environmental Microbiology *16*, 1472–1481.

D'Souza, G., Shitut, S., Preussger, D., Yousif, G., Waschina, S., and Kost, C. (2018). Ecology and evolution of metabolic cross-feeding interactions in bacteria. Natural Product Reports *35*, 455–488.

Emery, A.F., and Nenarokomov, A.V. (1998). Optimal experiment design. Meas. Sci. Technol. *9*, 864–876.

Egel, R. (1979) The *lac*-operon for lactose degradation, or rather for the utilization of galactosylglycerols from galactolipids? Journal of Theoretical Biology *79*, 117-119

Fazzino, L., Anisman, J., Chacón, J.M., Heineman, R.H., and Harcombe, W.R. (2020). Lytic bacteriophage have diverse indirect effects in a synthetic cross-feeding community. The ISME Journal *14*, 123–134

Förstner, K.U., Vogel, J., and Sharma, C.M. (2014). READemption—a tool for the computational analysis of deep-sequencing–based transcriptome data. Bioinformatics *30*, 3421–3423.

Fritts, R.K., McCully, A.L., and McKinlay, J.B. (2021). Extracellular metabolism sets the table for microbial cross-Feeding. Microbiology and Molecular Biology Reviews *85*, e00135-20.

Fussmann, G.F., Ellner, S.P., Shertzer, K.W., and Hairston Jr., N.G. (2000). Crossing the Hopf bifurcation in a live predator-prey system. Science *290*, 1358–1360.

Gadkar, K.G., Gunawan, R., and Doyle, F.J. (2005). Iterative approach to model identification of biological networks. BMC Bioinformatics *6*, 155.

Gardner, A., West, S.A., and Buckling, A. (2004). Bacteriocins, spite and virulence. Proceedings of the Royal Society of London. Series B: Biological Sciences *271*, 1529–1535.

Gillor, O., Giladi, I., and Riley, M.A. (2009). Persistence of colicinogenic *Escherichia coli* in the mouse gastrointestinal tract. BMC Microbiol *9*, 165.

Goetz, D.H., Holmes, M.A., Borregaard, N., Bluhm, M.E., Raymond, K.N., and Strong, R.K. (2002). The neutrophil lipocalin NGAL is a bacteriostatic agent that interferes with siderophore-mediated iron acquisition. Molecular Cell *10*, 1033–1043.

González-Pastor, J.E., Hobbs, E.C., and Losick, R. (2003). Cannibalism by sporulating bacteria. Science *301*, 510–513.

Granato, E.T., Meiller-Legrand, T.A., and Foster, K.R. (2019). The evolution and ecology of bacterial warfare. Current Biology *29*, R521–R537.

Gravesen, A., Jydegaard Axelsen, A.-M., Mendes da Silva, J., Hansen, T.B., and Knøchel, S. (2002). Frequency of Bacteriocin Resistance Development and Associated Fitness Costs in Listeria monocytogenes. Applied and Environmental Microbiology *68*, 756–764.

Griffin, A.S., West, S.A., and Buckling, A. (2004). Cooperation and competition in pathogenic bacteria. Nature *430*, 1024–1027.

Griggs, D.W., Tharp, B.B., and Konisky, J. (1987). Cloning and promoter identification of the iron-regulated *cir* gene of *Escherichia coli*. Journal of Bacteriology *169*, 5343–5352.

Hammarlund, S.P., Chacón, J.M., and Harcombe, W.R. (2019). A shared limiting resource leads to competitive exclusion in a cross-feeding system. Environmental Microbiology *21*, 759–771.

Hannah, R., Stroke, I., and Betz, N. (2003). Assay system: A luminescent β-galactosidase assay for multiple cell types and media. Cell Notes *6*, 16–18.

Harcombe, W. (2010). Novel cooperation experimentally evolved between species. Evolution *64*, 2166–2172.

Harcombe, W.R., Riehl, W.J., Dukovski, I., Granger, B.R., Betts, A., Lang, A.H., Bonilla, G., Kar, A., Leiby, N., Mehta, P., et al. (2014). Metabolic resource allocation in individual microbes determines ecosystem interactions and spatial dynamics. Cell Reports *7*, 1104–1115.

Harcombe, W. R., Chacón, J. M., Adamowicz, E. M., Chubiz, L. M., & Marx, C. J. (2018). Evolution of bidirectional costly mutualism from byproduct consumption. *Proceedings of the National Academy of Sciences*, *115*(47), 12000-12004.

Hawlena, H., Bashey, F., and Lively, C.M. (2012). Bacteriocin-mediated interactions within and between coexisting species. Ecology and Evolution *2*, 2516–2521.

Hibbing, M.E., Fuqua, C., Parsek, M.R., and Peterson, S.B. (2010). Bacterial competition: surviving and thriving in the microbial jungle. Nature Reviews Microbiology *8*, 15–25.

Hoek, T.A., Axelrod, K., Biancalani, T., Yurtsev, E.A., Liu, J., and Gore, J. (2016). Resource availability modulates the cooperative and competitive nature of a microbial cross-Feeding mutualism. PLOS Biology *14*, e1002540.

Hoiseth, S.K., and Stocker, B. a. D. (1981). Aromatic-dependent *Salmonella* typhimurium are non-virulent and effective as live vaccines. Nature *291*, 238–239.

Inglis, R.F., Scanlan, P., and Buckling, A. (2016). Iron availability shapes the evolution of bacteriocin resistance in *Pseudomonas aeruginosa*. The ISME Journal *10*, 2060–2066.

Jakes, K.S., and Cramer, W.A. (2012). Border crossings: Colicins and transporters. Annual Review of Genetics *46*, 209–231.

Kerr, B., Riley, M.A., Feldman, M.W., and Bohannan, B. (2002). Local dispersal promotes biodiversity in a real-life game of rock–paper–scissors. Nature *418*, 171–174.

Khare, A., and Tavazoie, S. (2015). Multifactorial competition and resistance in a two-species bacterial system. PLOS Genetics *11*, e1005715.

Kirkup, B.C., and Riley, M.A. (2004). Antibiotic-mediated antagonism leads to a bacterial game of rock–paper–scissors. Nature *428*, 412.

Kramer, J., Özkaya, Ö., and Kümmerli, R. (2020). Bacterial siderophores in community and host interactions. Nat Rev Microbiol *18*, 152–163.

Leisner, J.J., and Haaber, J. (2012). Intraguild predation provides a selection mechanism for bacterial antagonistic compounds. Proceedings of the Royal Society B *279*, 4513–4521.

Lewis, M. (2005). The lac repressor. Comptes Rendus Biologies *328*, 521–548.

Liu, J. Y., Miller, P. F., Willard, J., & Olson, E. R. (1999). Functional and biochemical characterization of Escherichia coli sugar efflux transporters. *The Journal of biological chemistry*, *274*(33), 22977–22984.

Majeed, H., Gillor, O., Kerr, B., and Riley, M.A. (2011). Competitive interactions in *Escherichia coli* populations: the role of bacteriocins. The ISME Journal *5*, 71–81.

Marcoleta, A.E., Gutiérrez-Cortez, S., Hurtado, F., Argandoña, Y., Corsini, G., Monasterio, O., and Lagos, R. (2018). The Ferric uptake regulator (Fur) and iron availability control the production and maturation of the antibacterial peptide microcin E492. PLOS ONE *13*, e0200835.

Nedialkova, L.P., Denzler, R., Koeppel, M.B., Diehl, M., Ring, D., Wille, T., Gerlach, R.G., and Stecher, B. (2014). Inflammation fuels colicin Ib-dependent competition of *Salmonella* serovar Typhimurium and *E. coli* in Enterobacterial blooms. PLOS Pathogens *10*, e1003844.

Nedialkova, L.P., Sidstedt, M., Koeppel, M.B., Spriewald, S., Ring, D., Gerlach, R.G., Bossi, L., and Stecher, B. (2016). Temperate phages promote colicin-dependent fitness of *Salmonella enterica* serovar Typhimurium. Environmental Microbiology *18*, 1591–1603.

Niehaus, F., Hantke, K., and Unden, G. (1991). Iron content and FNR-dependent gene regulation in *Escherichia coli*. FEMS Microbiology Letters *84*, 319–324.

Niehus, R., Picot, A., Oliveira, N.M., Mitri, S., and Foster, K.R. (2017). The evolution of siderophore production as a competitive trait. Evolution *71*, 1443–1455.

Palmer, J.D., and Foster, K.R. (2022) The evolution of spectrum in antibiotics and bacteriocins. PNAS *119*, e2205407119.

van der Ploeg, J.R. (2005). Regulation of bacteriocin production in *Streptococcus mutans* by the quorum-sensing system required for development of genetic competence. Journal of Bacteriology *187*, 3980–3989.

Porwollik, S., Santiviago, C.A., Cheng, P., Long, F., Desai, P., Fredlund, J., Srikumar, S., Silva, C.A., Chu, W., Chen, X., et al. (2014). Defined single-gene and multi-gene deletion mutant collection in *Salmonella enterica* sv Typhimurium. PLOS ONE *9*, e99280.

Ratzke, C., and Gore, J. (2016). Self-organized patchiness facilitates survival in a cooperatively growing *Bacillus subtilis* population. Nature Microbiology *1*, 16022.

Riley, M.A., and Gordon, D.M. (1999). The ecological role of bacteriocins in bacterial competition. Trends in Microbiology *7*, 129–133.

Rishi, P., Woodward, C.L., Kim, W.-K., and Ricke, S.C. (2004). *Salmonella enterica* serovar Typhimurium *hilA-lacZY* fusion gene response to iron chelation or supplementation in rich and minimal media. Journal of Environmental Science and Health, Part B *39*, 861–870.

Salvail, H., Caron, M.-P., Bélanger, J., and Massé, E. (2013). Antagonistic functions between the RNA chaperone Hfq and an sRNA regulate sensitivity to the antibiotic colicin. The EMBO Journal *32*, 2764–2778.

Samuels, A.N., Roggiani, M., Smith, K.A., Zhu, J., Goulian, M., and Kohli, R.M. (2020). Deciphering the role of colicins during colonization of the mammalian gut by commensal *E. coli*. Microorganisms *8*, 664.

Semsey, S., Andersson, A., Krishna, S., Jensen, M.H., Massé, E., and Sneppen, K. (2006). Genetic regulation of fluxes: iron homeostasis of *Escherichia coli*. Nucleic Acids Research *34*, 4960–4967.

Simon, R., Priefer, U., and Pühler, A. (1983). A broad host range mobilization system for *in vivo* genetic engineering: transposon mutagenesis in Gram Negative bacteria. Nat Biotechnol *1*, 784–791.

Spriewald, S., Stadler, E., Hense, B.A., Münch, P.C., McHardy, A.C., Weiss, A.S., Obeng, N., Müller, J., and Stecher, B. (2020). Evolutionary stabilization of cooperative toxin production through a bacterium-plasmid-phage interplay. MBio *11*, e00912-20.

Stecher, B., Denzler, R., Maier, L., Bernet, F., Sanders, M.J., Pickard, D.J., Barthel, M., Wetendorf, A.M., Krogfelt, K.A., Walker, A.W., et al. (2012). Gut inflammation can boost horizontal gene transfer between pathogenic and commensal Enterobacteriaceae. Proc. Natl. Acad. Sci. USA *109*, 1269–1274.

Walker, D., Rolfe, M., Thompson, A., Moore, G.R., James, R., Hinton, J., and Kleanthous, C. (2004). Transcriptional profiling of colicin-induced cell death of Escherichia coli MG1655 identifies potential mechanisms by which bacteriocins promote bacterial diversity. Journal of Bacteriology *186*, 866–869.

West, S.A., Diggle, S.P., Buckling, A., Gardner, A., and Griffin, A.S. (2007). Social lives of microbes. Annual Review of Ecology, Evolution and Systematics *38*, 53–77.

Yao, K.Z., Shaw, B.M., Kou, B., McAuley, K.B., and Bacon, D.W. (2003). Modeling ethylene/butene copolymerization with multi-site catalysts: Parameter estimability and experimental design. Polymer Reaction Engineering *11*, 563–588.

Zhou, K., Qiao, K., Edgar, S., and Stephanopoulos, G. (2015). Distributing a metabolic pathway among a microbial consortium enhances production of natural products. Nat Biotechnol *33*, 377–383.
